# Comprehensive transcriptome and methylome analysis delineates the biological basis of hair follicle development and wool-related traits in Merino sheep

**DOI:** 10.1186/s12915-021-01127-9

**Published:** 2021-09-09

**Authors:** Bingru Zhao, Hanpeng Luo, Junmin He, Xixia Huang, Siqian Chen, Xuefeng Fu, Weidan Zeng, Yuezhen Tian, Shuli Liu, Cong-jun Li, George E. Liu, Lingzhao Fang, Shengli Zhang, Kechuan Tian

**Affiliations:** 1 National Engineering Laboratory for Animal Breeding, Key Laboratory of Animal Genetics, Breeding, and Reproduction, Ministry of Agriculture, College of Animal Science and Technology, China Agricultural University, Beijing, China; 2 Key Laboratory of Genetics Breeding and Reproduction of the Fine Wool Sheep & Cashmere Goat in Xinjiang, Institute of Animal Science, Xinjiang Academy of Animal Sciences, Urumqi, China; 3 College of Animal Science, Xinjiang Agricultural University, Urumqi, China; 4 Animal Genomics and Improvement Laboratory, Henry A. Wallace Beltsville Agricultural Research Center, Agricultural Research Service, Agricultural Research Service, USDA, Beltsville, Maryland USA; 5 MRC Human Genetics Unit at the Institute of Genetics and Cancer, University of Edinburgh, Edinburgh, UK; 6 Institute of Animal Science and Veterinary Medicine, Shandong Academy of Agricultural Sciences, Jinan, China

**Keywords:** Developmental stage, DNA methylation, Genome-wide association study, Hair follicle morphogenesis, Sheep, Transcriptome

## Abstract

**Background:**

Characterization of the molecular mechanisms underlying hair follicle development is of paramount importance in the genetic improvement of wool-related traits in sheep and skin-related traits in humans. The Merino is the most important breed of fine-wooled sheep in the world. In this study, we systematically investigated the complexity of sheep hair follicle development by integrating transcriptome and methylome datasets from Merino sheep skin.

**Results:**

We analysed 72 sequence datasets, including DNA methylome and the whole transcriptome of four gene types, i.e. protein-coding genes (PCGs), lncRNAs, circRNAs, and miRNAs, across four embryonic days (E65, E85, E105, and E135) and two postnatal days (P7 and P30) from the skin tissue of 18 Merino sheep. We revealed distinct expression profiles of these four gene types across six hair follicle developmental stages, and demonstrated their complex interactions with DNA methylation. PCGs with stage-specific expression or regulated by stage-specific lncRNAs, circRNAs, and miRNAs were significantly enriched in epithelial differentiation and hair follicle morphogenesis. Regulatory network and gene co-expression analyses identified key transcripts controlling hair follicle development. We further predicted transcriptional factors (e.g. KLF4, LEF1, HOXC13, RBPJ, VDR, RARA, and STAT3) with stage-specific involvement in hair follicle morphogenesis. Through integrating these stage-specific genomic features with results from genome-wide association studies (GWAS) of five wool-related traits in 7135 Merino sheep, we detected developmental stages and genes that were relevant with wool-related traits in sheep. For instance, genes that were specifically upregulated at E105 were significantly associated with most of wool-related traits. A phenome-wide association study (PheWAS) demonstrated that candidate genes of wool-related traits (e.g. *SPHK1*, *GHR*, *PPP1R27*, *CSRP2*, *EEF1A2*, and *PTPN1*) in sheep were also significantly associated with dermatological, metabolic, and immune traits in humans.

**Conclusions:**

Our study provides novel insights into the molecular basis of hair follicle morphogenesis and will serve as a foundation to improve breeding for wool traits in sheep. It also indicates the importance of studying gene expression in the normal development of organs in understanding the genetic architecture of economically important traits in livestock. The datasets generated here are useful resources for functionally annotating the sheep genome, and for elucidating early skin development in mammals, including humans.

**Supplementary Information:**

The online version contains supplementary material available at 10.1186/s12915-021-01127-9.

## Background

In mammals, hair follicles are crucial for temperature regulation, physical protection, sweat and sebum dispersion, sensory and tactile functions, and social interactions [1, 2]. The hair follicle morphogenesis in sheep is often divided into five stages, including induction (embryonic day 65, E65), organogenesis (E85, primary and secondary follicles related), differentiation (E105 ~ E135, especially for secondary follicles), maturation (postnatal day 7, P7), and postnatal hair cycle stages (P30) [3, 4]. The number of dermal papilla cells and the size of hair placode are associated with the diameter, crimp, and density of wool fibres [3], which are of high economic value in sheep industry. In addition, the normal development of hair follicle highly interacts with the immune system, such as the immune capacity of hair follicle [5–7]. The protection of hair follicles and their stem cells against the autoimmune system is fundamental to guarantee the normal development of hair follicles [3, 8]. Similar to head hair follicles in humans, the occurrence and growth of hair follicles in sheep are independent from neighbouring follicles. The sweat glands and wool follicles as unique features of adult sheep trunk skin resemble those in human axillary skin [9]. Therefore, the characterization of molecular mechanisms underlying the hair follicle morphogenesis in sheep will help understand the genetic basis of wool-related traits and can serve as a valuable model for skin-relevant diseases in humans.

The normal morphogenesis and development of hair follicle is the culmination of spatiotemporal expression of genes under the control of genetic and epigenetic elements, such as long non-coding RNA (lncRNA), microRNAs (miRNAs), circular RNAs (circRNAs), and DNA methylation [10, 11]. Although several studies have investigated the development of skin in sheep previously, they were limited in terms of developmental stages and gene types. For instance, Nie et al. [4] explored the global changes of lncRNAs and mRNAs at two developmental stages during the induction of primary wool follicles in Carpet sheep. Zhao et al. [12] investigated the involvement of lncRNA-miRNA-mRNA interaction networks in the hair follicle induction across three developmental stages in Aohan sheep.

The majority of genetic variants discovered in genome-wide association studies (GWAS) are non-coding [13]. Better characterization of the regulatory elements in the livestock genome, such as through the efforts of the ongoing Functional Annotation of Animal Genomes (FAANG) and the Farm animal Genotype-Tissue Expression (FarmGTEx) projects [14–16], is therefore essential for biologically interpreting the GWAS loci of complex traits of economic value. Furthermore, integrating functional annotations that are specific to tissues and developmental stages with GWAS results can help reveal the causal tissues, developmental stages, and genes for complex traits and diseases [17–19].

In this study (Additional file 1: Fig. S1), to comprehensively characterize the molecular mechanisms underpinning hair follicle development in sheep, we sequenced DNA methylation and the whole transcriptome of four gene types, including protein-coding genes (PCGs), miRNAs, circRNAs, and lncRNAs, across six important hair follicle developmental stages (i.e. E65, E85, E105, E135, P7, and P30) in the skin tissue of 18 Merino sheep. In total, we newly generated 72 sequence datasets, which enabled us to identify the key genes, transcriptional factors (TFs), signalling pathways, and interaction networks regulating hair follicle morphogenesis across developmental stages. We then integrated these multi-molecular features specific to each developmental stage with GWAS signals of five wool-related traits and one growth trait to understand the genetic and biological basis of such traits in sheep. These traits included mean fibre diameter (MFD), coefficient of variation of the fibre diameter (CVFD), crimp number (CN), mean staple length (MSL), greasy fleece weight (GFW), and live weight (LW) in 7135 Merino sheep. The resources and findings generated here will provide an opportunity to better annotate the sheep genome by predicting novel non-coding genes and inferring the function of unannotated genes *via* co-expression networks, as well as to enhance the genetic improvement of wool traits in sheep.

## Results

### Histological changes of hair follicles across developmental stages

**Fig. 1:**
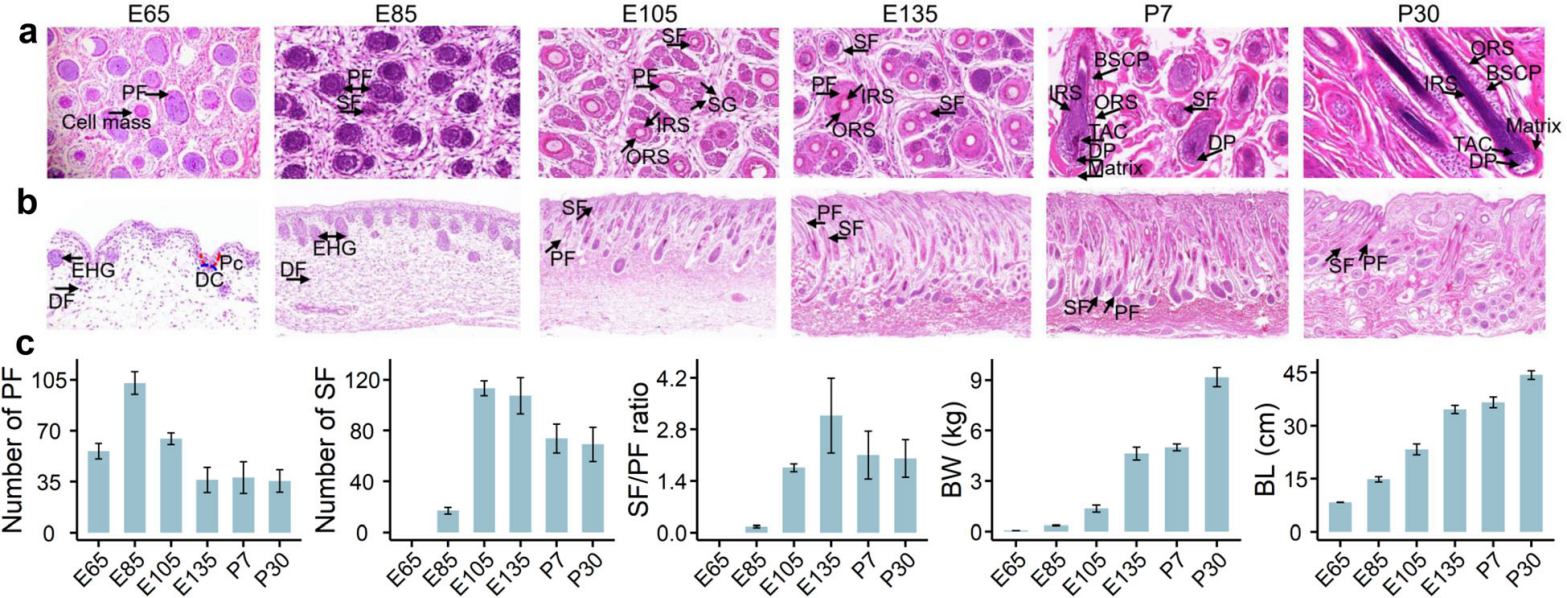
Morphological observation of hair follicles across six developmental stages in sheep. **a, b** Haematoxylin-eosin (H&E) staining of skin for horizontal (magnification: × 40) and longitudinal (× 10) sections at embryonic day 65 (E65), E85, E105, E135, and postnatal day 7 (P7) and P30, respectively. The induction of hair follicles is initiated around E65 and characterized by crowded epidermal keratinocytes, hair placode (Pc) (red dashed lines) and dermal condensate (DC) (blue dashed lines). At E85, the number of primary follicles (PFs) increases significantly, and secondary follicles (SFs) start to form. At E105, PFs are regularly arranged, and the surrounding SFs, sebaceous gland (SG), outer root sheath (ORS), and inner root sheath (IRS) layers are clearly visible. At E135, the secondary-derived follicles branching from SFs appear. At P7, hair follicles mature with complete structure. The dermal papilla (DP), ORS, and IRS structures are clearly visible, and hair shafts emerge through the epidermis. At P30, the hair follicles enter into the mature anagen phase. DF, dermal fibroblast; EHG, elongated hair germ; TAC, transit amplifying cells; BSCP, bulge stem cell precursors. **c** Numbers of PF and SF, SF/PF ratio, body weight and length (average of three replicates; mean ± SD) of Merino sheep during hair follicles development

We performed the haematoxylin-eosin (H&E) staining for horizontal and longitudinal sections of skin to observe the histological changes of hair follicles across six developmental stages (i.e. E65, E85, E105, E135, P7, and P30) (Fig. 1a, b). The hair placode (Pc) and dermal condensate (DC) started to form at E65, indicating the induction of hair follicles. At E85, the number of primary follicles (PFs) increased and the secondary follicles (SFs) started to form. At E105, the SFs started to differentiate, and the number of secondary-derived follicles increased at E135. At P7, hair follicles matured with a complete structure, and hair shafts emerged through the epidermis. At P30, hair follicles entered into the anagen phase, during which the root of the hair divides rapidly, adding to the hair shaft. The numbers of PF and SF, the SF/PF ratios (an indicator of wool fineness), and the body weights and lengths through all these six stages are shown in Fig. 1c. In general, the numbers of PF and SF peaked at E85 and E105, respectively, and as expected, body weights (BW) and body lengths (BL) increased across developmental stages.

### Identification of stage-specific transcripts and DNA methylation regions during hair follicle development

In total, we generated 72 distinct RNA sequencing datasets for PCGs, lncRNAs, circRNAs, and miRNAs across six developmental stages in 18 animals, producing 3,998,197,856 clean reads with an average mapping rate of 90.46%. We also generated 18 methylated DNA immunoprecipitation sequencing (MeDlP-Seq) datasets, yielding 1,028,125,898 clean reads with an average mapping rate of 97.19%. The mapping details of all the 72 datasets are described in Additional file 2: Table S1.

We quantified and normalized the expression levels of four gene types across samples, including 19,229 PCGs, 10,193 lncRNAs (1540 existing and 8653 novel), 151 known miRNAs (98.7% existing in the miRBase database (Release 22.1) [20]), and 41,369 novel circRNAs (Additional file 3: Table S2). We identified a total of 1,730,765 methylation peaks in the 18 samples, and the number of peaks across major genomic features (e.g. 5′UTR, exon and 3′UTR) in each sample is summarized in Additional file 4: Table S3.

**Fig. 2:**
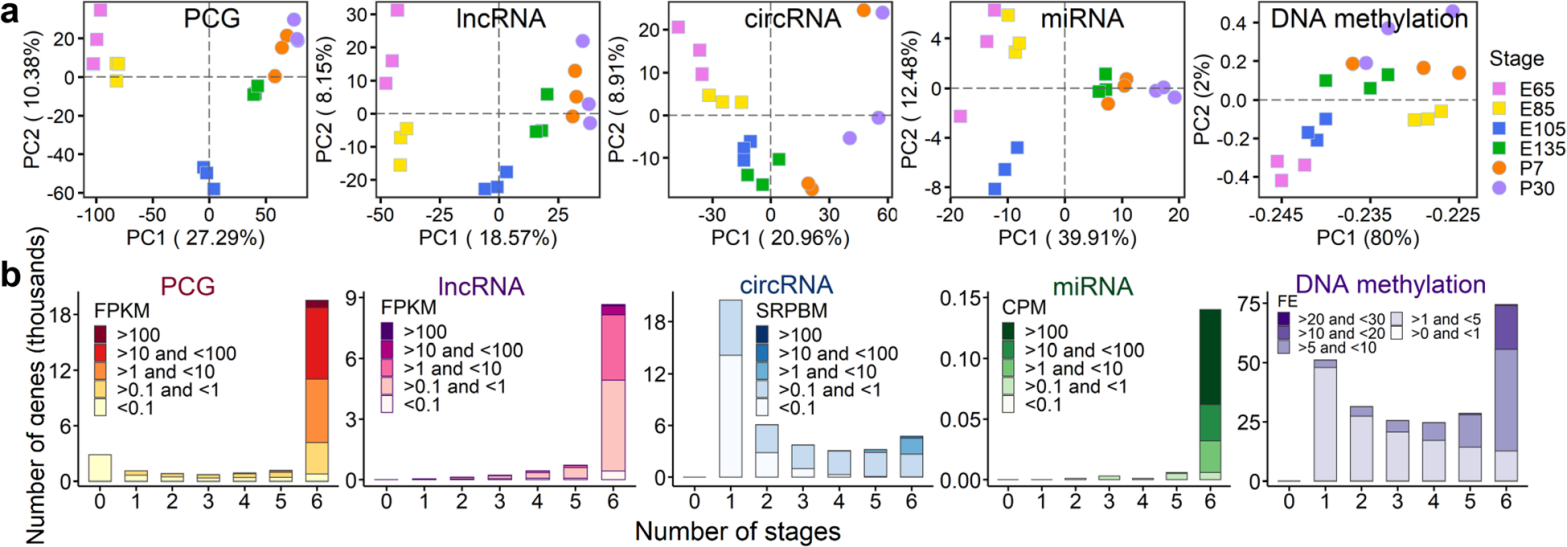
General characteristics of molecular features in sheep skin tissue across developmental stages. **a** Principal component analysis (PCA) of all 18 samples based on the expression levels of four gene types and DNA methylation. PCG, protein-coding gene. **b** The distribution of gene expression and DNA methylation across number of stages. FPKM, fragments per kilobase of exon model per million mapped fragments; SRPBM, spliced reads per billion mapping; CPM, counts per million; FE, fold enrichment

The principal component analysis (PCA) of all 18 samples based on the five molecular profiles consistently revealed that the developmental stage was the major factor distinguishing samples (Fig. 2a, Additional file 5: Fig. S2). Distributions of the expression levels of four gene types and DNA methylation levels across developmental stages are shown in Fig. 2b. Overall, the majority of PCGs, lncRNAs, and miRNAs were ubiquitously expressed across all six stages, while circRNAs showed clear stage-specific expression. We found that all circRNAs were derived from 6477 parental genes, with the majority of (27,233 out of 41,369) them from coding DNA sequences (CDS) (Additional file 6: Fig. S3a). Of these parental genes, 4550 produced more than one circRNAs (Additional file 6: Fig. S3b), suggesting that circRNAs are abundant during skin development. Furthermore, we explored the stage specificity of PCGs, lncRNAs, circRNAs, miRNAs, and methylated regions (MRs) during hair follicle morphogenesis. The greatest number of stage-specific transcripts was detected at E65 (the induction of hair follicle) and at P30 (the anagen of hair follicle) (Additional file 7: Fig. S4), which was in line with the development of hair follicles (Fig. 1a–c).

### Dynamic expression patterns of PCGs during the hair follicle morphogenesis

**Fig. 3.**
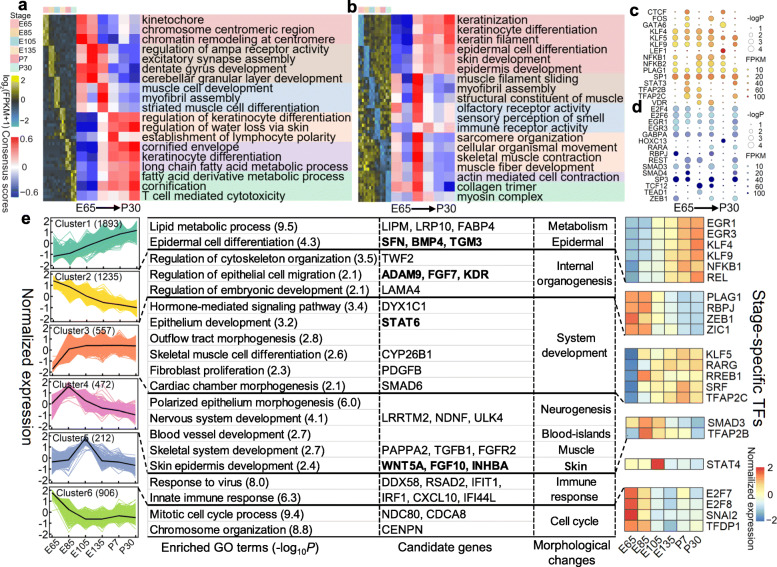
Dynamic expression patterns of protein-coding genes (PCGs) during hair follicle development. **a** Heatmaps (left) shows the expression level (log_2_(FPKM+ 1)) of top 10 upregulated stage-specific PCGs across developmental stages; heatmaps (right) shows the normalized consensus scores of significantly (FDR < 0.05) enriched Gene Ontology (GO) terms for all upregulated genes at each stage by the gene set enrichment analysis (GSEA). FPKM, fragments per kilobase of exon model per million mapped fragments. **b** Similar to **a**, but for the downregulated stage-specific PCGs. **c, d** Motifs of transcriptional factors (TFs) are significantly enriched in promoters of upregulated and downregulated stage-specific PCGs, respectively. **e** K-means clusters of all stage-specific PCGs. Genes in bold are those involved in the skin and epithelium development. Corresponding biological themes and the top enriched TF motifs are shown next to each cluster

The gene set enrichment analysis (GSEA) of stage-specific PCGs revealed significantly (FDR < 0.05) enriched Gene Ontology (GO) terms, which were distinct across developmental stages (Fig. 3a, b, Additional file 8: Table S4). For instance, the PCGs upregulated at E65, E85, E105, E135, P7, and P30 were significantly enriched in chromosome assembly, neurological system, muscle development, regulation of keratinocyte differentiation, keratinocyte differentiation, and lipid metabolism, respectively. The upregulated PCGs at E135-P30 were also significantly enriched in various immune processes such as lymphocyte polarity establishment and T cell-mediated cytotoxicity (Fig. 3a). The downregulated PCGs at E105 were significantly enriched in muscle development and sensory perception of stimuli, while the downregulated PCGs at E135-P30 were significantly enriched in the keratinocyte and epidermal cell differentiation, skin development, and immune processes (Fig. 3b).

To investigate whether PCGs with stage-specific expression were collectively regulated by certain TFs, we performed a motif enrichment analysis on the promoters (1500 bp up- and 500 bp downstream of transcription start sites, TSS) of upregulated and downregulated stage-specific PCGs separately. We detected 29 and 27 significant motifs (FDR < 0.05) for upregulated and downregulated PCGs, respectively, mainly including the KLF (*n* = 33), EGR (*n* = 32), and SOX (*n* = 17) motif families (Additional file 9: Table S5). We found that the enrichment of motifs and the expression of their target TFs were stage-specific, indicating that these TFs might play vital roles during embryo and hair follicle development (Fig. 3c, d). For instance, PLAG1, EGR1, EGR3, GATA6, and TFAP2C are essential for embryonic organ formation [21–23], while KLF4, LEF1, HOXC13, RBPJ, VDR, RARA, and STAT3 are crucial for hair follicle differentiation and development [24–30]. We further found that NFKB1, NFKB2, and IRF1, which participate in the immune function and inflammation [31], were significant at E105 and E135.

We grouped all stage-specific PCGs into six clusters with different expression trends across stages, and also found that these clusters exhibited distinct biological functions and motif enrichment patterns (Fig. 3e). For instance, PCGs in Cluster1, whose expression levels gradually increased across developmental stages, were significantly (*P* < 0.01) enriched in epidermal cell differentiation and lipid metabolism. The promoters of these PCGs were also significantly enriched for early growth-specific TF motifs, such as EGR3 [32] and KLF4 [33]. In contrast to Cluster1, we observed that expression levels of PCGs in Cluster2 gradually decreased across developmental stages. These genes were significantly enriched in internal organogenesis, and their promoters were significantly enriched for organogenesis-related TFs (e.g. ZEB1) [34] and notch signalling pathway-related TFs (e.g. RBPJ) [27]. The PCGs in Cluster5 showed the highest expression levels at E105 and were significantly (*P* < 0.01) enriched in the immune response. Their promoters were significantly enriched for motifs of STAT4, which plays a key role in the immune system [35]. The expression patterns of enriched TFs were consistent with the enrichment of their motifs across developmental stages (Fig. 3e), providing more evidence that these TFs paly crucial regulatory roles in gene expression during normal skin development.

### Stage-specific regulatory mechanisms of non-coding RNAs and DNA methylation

**Fig. 4:**
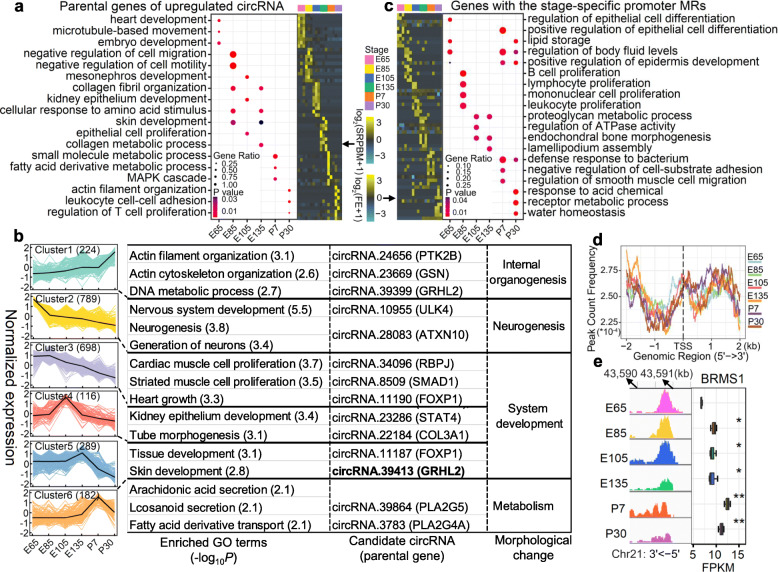
Stage-specific regulatory mechanisms of circRNAs and DNA methylation. **a** Heatmap shows the expression (log_2_(SRPBM+ 1)) of upregulated stage-specific circRNAs across developmental stages, and the bubble plot shows the top three enriched gene ontology (GO) terms for parental genes of the upregulated circRNAs across stages. **b** K-means clustering of all stage-specific circRNAs. Corresponding biological processes are shown next to each cluster. **c** Heatmap shows the DNA methylation signals (log_2_(FE + 1)) of stage-specific methylated regions (MRs) in promoters (1500 bp upstream and 500 bp downstream of transcriptional start sites, TSSs). FE, fold enrichment. The bubble plot shows the top five enriched gene ontology (GO) terms for genes with stage-specific MRs in promoter. **d** Distribution of DNA methylation levels around TSSs across all six skin developmental stages. **e** The methylation level of *BRMS1* promoter decreases, whereas its expression level increases across developmental stages

To investigate the roles of circRNAs in the gene regulation during hair follicle development, we performed a GO enrichment analysis for parental genes of stage-specific circRNAs. The parental genes of upregulated circRNAs participated in embryo development at E65, skin and cellular development at E85, system development and epithelial cell proliferation at E105, skin development at E135, metabolic process at P7, and immune response at P30 (Fig. 4a). The parental genes of downregulated circRNAs were significantly (*P* < 0.01) enriched in skin development at E65, protein catabolism at E85, immune response at E105, and central nervous system (CNS) at E135 to P30 (Additional file 10: Fig. S5). We further divided the stage-specific circRNAs into six clusters according to their expression trends across the developmental stages (Fig. 4b). The expression levels of 224 circRNAs in Cluster1 increased across developmental stages, whose parental genes were significantly enriched in internal organogenesis. Conversely, cluster2 comprised of 789 circRNAs with developmentally decreased expression levels, whose parental genes were significantly enriched in neurogenesis. Meanwhile, the 289 circRNAs in cluster 5 demonstrated the highest expression levels at E135, whose parental genes were significantly enriched in skin development, such as circRNA.39413 (*GRHL2*) (Fig. 4b).

Furthermore, we explored the biological functions of genes that were regulated by lncRNAs and miRNAs across developmental stages. For instance, targets of both upregulated and downregulated lncRNAs were significantly enriched in immune and metabolic processes (Additional file 11: Fig. S6a, b). The targets of upregulated stage-specific miRNAs were significantly enriched in homeostasis-related at E65, internal organogenesis at E85, cellular developmental process at E105, and hormone secretion-related at P30 (Additional file 11: Fig. S6c). The targets of downregulated stage-specific miRNAs were significantly enriched in the hormone secretion at E85, metabolic process at P30, and notch signalling pathway at the rest of stages (Additional file 11: Fig. S6d). Overall, these results indicated that non-coding RNAs play important roles in skin development by distinctly regulating the expression of their target genes across developmental stages.

The functional enrichment analysis revealed that genes with the stage-specific promoter MRs were significantly enriched in epithelial cell differentiation at E65 and P7, and in immune processes at E85 (Fig. 4c). In general, we observed that the upstream DNA methylation levels of TSSs were lower at the later stages than the earlier ones (Fig. 4d). We took *BRMS1* as an example in Fig. 4c, which participates in the epidermal growth factor receptor (EGFR) and NF-κB signalling pathways [36]. The promoter methylation level of *BRMS1* decreased, while its expression level increased across developmental stages.

### Regulatory networks of PCGs and non-coding RNAs

**Fig. 5:**
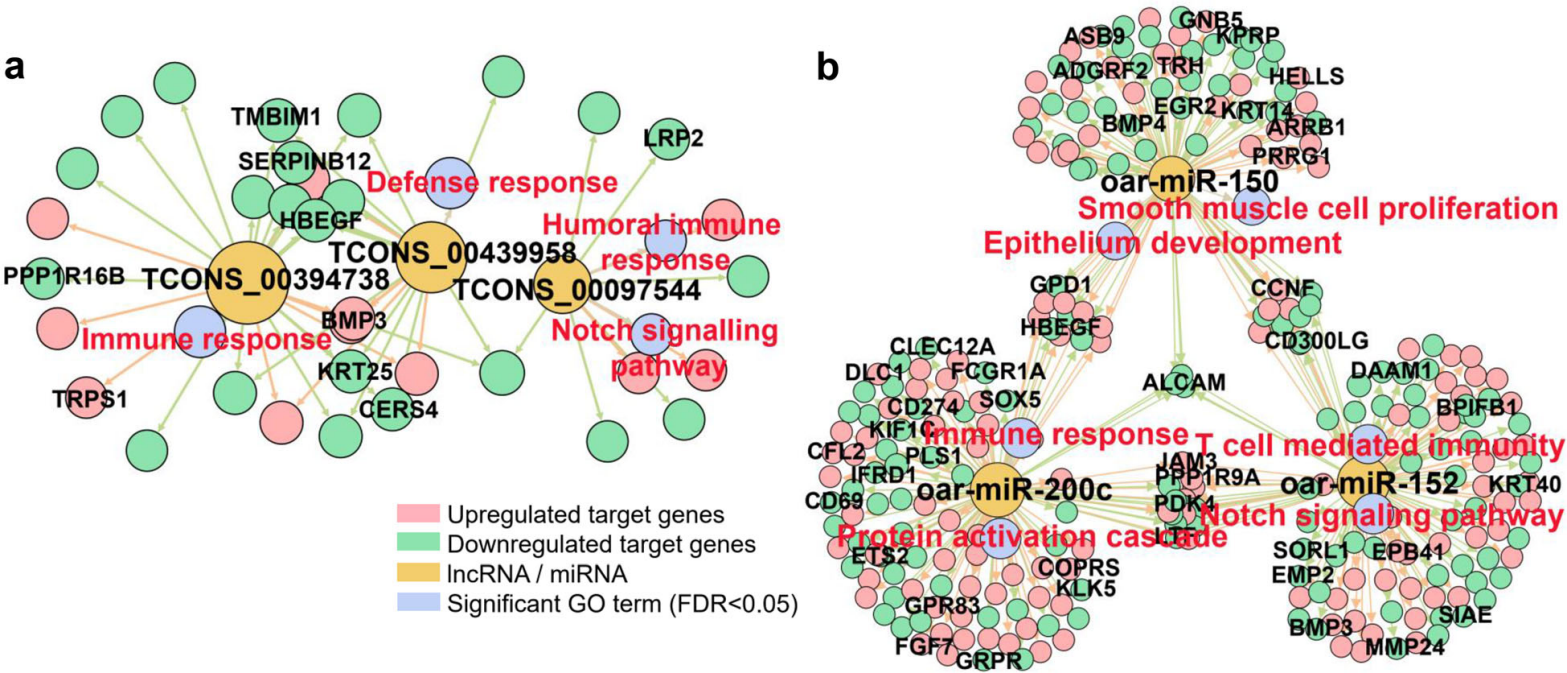
The regulatory networks of target genes of non-coding RNAs. **a** The regulatory networks of targets of three lncRNAs, i.e. TCONS_00394738, TCONS_00439958, and TCONS_00097544. **b** The regulatory networks of targets of three miRNAs, i.e. oar-miR-150, oar-miR-200c, and oar-miR-152. The colours of the edges are the directions (pink for upregulated; green for downregulated) of lncRNAs and miRNAs regulating target genes. The sizes of the circles correspond to the interaction degrees

We predicted the target genes for each miRNA and lncRNA, and further investigated whether the stage-specific PCGs at each developmental stage were highly enriched for targets of certain miRNAs and lncRNAs. If this was the case, then the enriched miRNAs and lncRNAs might be implicated in skin development. The detailed summary statistics are listed in Additional file 12: Table S6. For instance, TCONS_00394738, TCONS_00439958, and TCONS_00097544 were the top lncRNAs, whose targets were significantly (FDR < 0.05) enriched for PCGs with specific expression at E85. The targets of these three lncRNAs were significantly (FDR < 0.05) engaged in immune response and the Notch signalling pathway (Fig. 5a). The targets of the top three significant (FDR < 0.05) miRNAs at E65, Oar-miR-150, oar-miR-200c, and oar-miR-152, were significantly (FDR < 0.05) enriched in epithelial development, immune response, and the Notch signalling pathway (Fig. 5b).

**Fig. 6:**
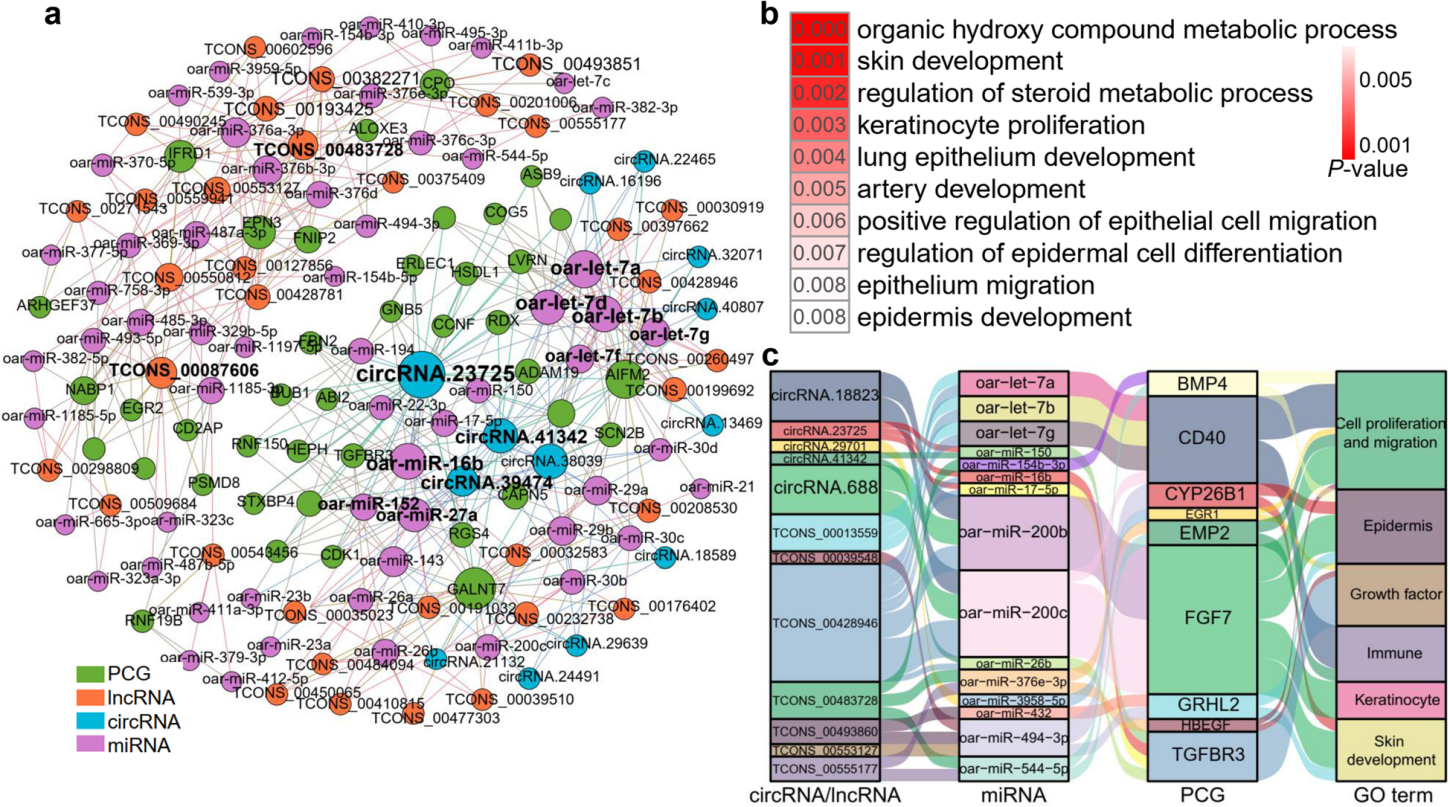
Competing endogenous RNA (ceRNA) network. **a** The top 200 circRNA-miRNA-mRNA and top 200 lncRNA-miRNA-mRNA interactions. The edges are the Pearson correlation coefficient (PCC) between genes. The sizes of the circles correspond to the connection degrees. **b** Top10 enriched Gene Ontology (GO) terms (biological processes, BP) in ceRNAs comparing to the genome background. The *P* values are computed by the hypergeometric test. **c** Sankey diagram of important candidate ceRNA pairs. Each rectangle represents a gene; degree of connection of each gene is directly proportional to rectangle size

We further performed a competing endogenous RNA (ceRNA) analysis to detect the regulatory effects of circRNAs or lncRNAs on the expression of PCGs by mediating miRNAs during hair follicle morphogenesis. A detailed summary of the negative correlation (Pearson correlation coefficient (PCC) < − 0.7) of miRNA-target pairs is in Additional file 13: Table S7, while the positive correlation (PCC > 0.7) of ceRNA pairs (lncRNA-PCG and circRNA-PCG) which were targeted by a common miRNA (the hypergeometric test, *P* < 0.05) is in Additional file 14: Table S8. We displayed the top 200 circRNA-miRNA-mRNA and the top 200 lncRNA-miRNA-mRNA interactions in Fig. 6a. The GO functional enrichment analysis revealed that genes in this ceRNA network were significantly enriched in skin development, keratinocyte proliferation, epidermal development (Fig. 6b). We proposed 12 most promising circRNAs and lncRNAs that acted as ceRNAs to affect the expression of stage-specific PCGs by sponging miRNAs during hair follicle morphogenesis (Fig. 6c). For instance, *FGF7* exhibited the specific expression at E65 (Fig. 3e), which participates in skin development, keratinocyte proliferation, and cell proliferation. The expression of *FGF7* was regulated by circRNA.18823, circRNA.688, and TCONS_00428946 through mediating oar-miR-200b and oar-miR-200c (Fig. 6c). Similarly, the expression of *GRHL2* was regulated by TCONS_000493860 through mediating oar-miR-1197-3p, oar-miR-432, and oar-miR-494-3p. *GRHL2* was specifically expressed at E85 and regulates epidermal cell differentiation and skin development [37] (Fig. 6c, Fig. 3e). Altogether, these results shed light on the complex interaction networks of PCGs and non-coding RNAs, which regulate hair follicle differentiation and growth.

### Gene co-expression network analysis

**Fig. 7:**
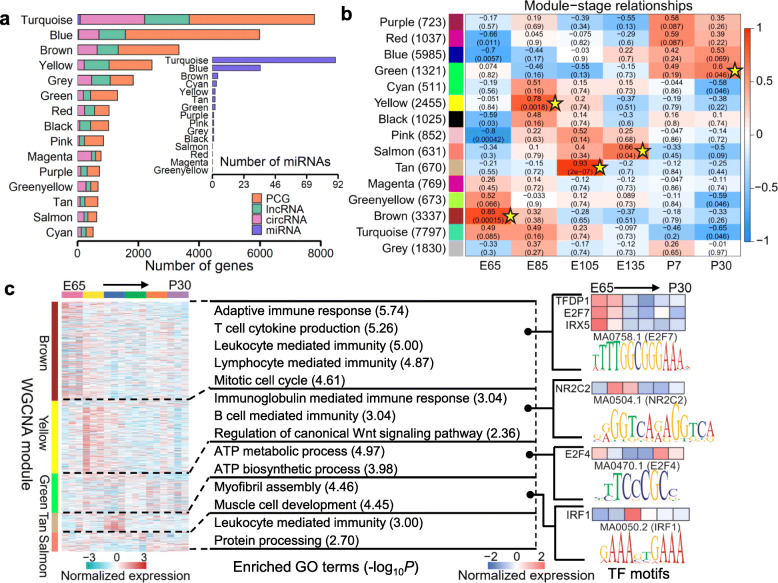
The weighted gene co-expression network analysis (WGCNA). **a** Bar chart shows numbers of each gene type detected in individual modules. Grey module includes genes that are not assigned to any co-expression modules. **b** Correlations between gene modules and developmental stages. The statistical significance of module-developmental stage relationship is corrected for multiple testing using the FDR method. The yellow stars denote FDR < 0.05. Each cell contains the correlation and the corresponding FDR value in bracket. **c** Heatmap shows the normalized gene expression for genes in the top five significant modules. Top enriched GO terms (biological process; BP), the normalized gene expression for module-enriched TFs, and the top representative sequence motif are shown next to each module

We performed an unsigned weighted gene co-expression network analysis (WGCNA) among PCG, lncRNA, circRNA, and miRNA to identify co-expression modules related to hair follicle morphogenesis. In total, we detected 15 co-expression modules with the four gene types (Fig. 7a). The expression patterns of five modules were significantly (FDR < 0.5) stage-specific (Fig. 7b). The GO functional enrichment analyses revealed that these stage-specific modules were significantly enriched in immune response, cell cycle phase, and metabolic process (Fig. 7c, Additional file 15: Fig. S7a). In addition, we found that many genes without functional annotation were co-expressed with functionally annotated genes (Additional file 15: Fig. S7 b, c). For instance, there were 1135 genes (347 PCGs, 635 lncRNAs, 153 circRNAs) with no functional information co-expressed with other annotated genes in a module (coloured brown), which were significantly enriched in the immunity system. These results indicated that our newly generated datasets can serve as a useful resource for functionally annotating genes in sheep.

We detected six TFs, whose motifs were significantly enriched in these five stage-specific modules (Fig. 7c). These TFs also showed stage-specific expression and participate in embryonic development. For instance, TFDP1 and E2F4 participate in the TGF-β signalling pathway [38] and showed stage-specific expression at E65 and P7 (Fig. 7c). IRF1 serves as an activator of genes involved in the innate and acquired immune responses. It also enhanced the expression of interferon-kappa (IF-κ) [39] and showed stage-specific expression at E105 (Fig. 7c).

### Integrative analysis of stage-specific molecular features with GWAS signals of wool and growth traits

**Fig. 8.**
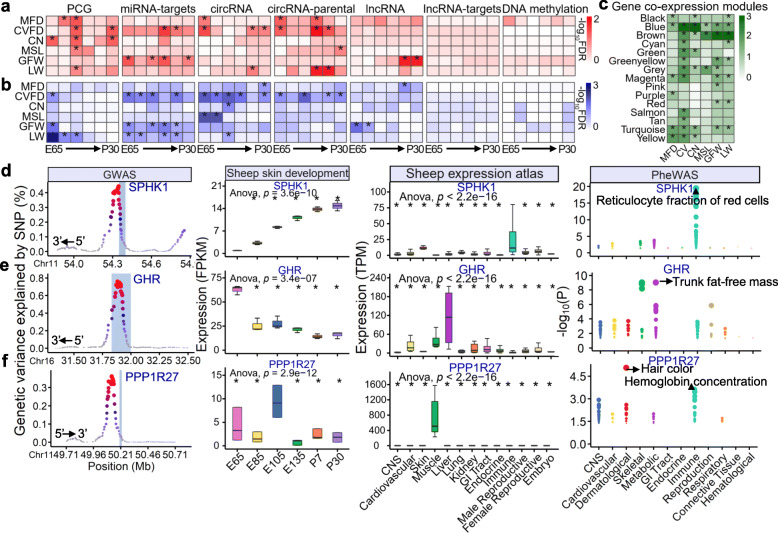
Integrative analysis of stage-specific molecular signatures and co-expression modules with GWAS signals of wool and growth traits. **a** The GWAS signal enrichment results across the stage-specific upregulated molecular features including PCGs, miRNA targets, circRNAs, circRNAs parental genes, lncRNAs, lncRNA targets, and DNA methylation. **b** Similar to **a**, but for stage-specific downregulated molecular features. **c** The GWAS signal enrichment results for 15 gene co-expression modules. Traits include mean fibre diameter (MFD), coefficient of variation of fibre diameter (CVFD), crimp number (CN), mean staple length (MSL), greasy fleece weight (GFW), and live weight (LW). Colour corresponds to enrichment degrees (− log_10_FDR) calculated using the sum-based GWAS signal enrichment analysis, where (∗) means FDR < 0.1. **d**, **e** *GHR*; **f** *PPP1R27*. For left to right, the first plot is for the genetic variance explained by SNPs of *SPHK1* (each dot is one SNP, and the highlighted region corresponds to gene region), the second for the expression patterns of *SPHK1* across six developmental stages, the third for the expression patterns of *SPHK1* across multiple tissues in sheep gene expression atlas, and the last for the results of phenome-wide association study (PheWAS) for *SPHK1*. **e**, **f** Similar to **d**, but for *GHR* and *PPP1R27*, respectively

To determine whether the developmental gene expression and regulation patterns allow us to better interpret the genetic variants associated with complex traits, we integrated the stage-specific molecular features detected above with GWAS signals of five wool traits and live weight in Merino sheep (Additional file 16: Table S9) [40]. As shown in Fig. 8a, b, genes with stage-specific expression were significantly (FDR < 0.1) enriched for GWAS signals of all these traits. For instance, genes with specific upregulation at E105 were significantly (FDR < 0.1) enriched in GWAS signals of five traits, including MFD, CVFD, CN, MSL, and LW (Fig. 8a). In addition, targets of miRNAs and circRNAs, which showed specific upregulation at E135 and P7, were significantly (FDR < 0.1) enriched for GWAS signals of CVFD. Targets of lncRNAs and miRNAs with P30 specific upregulation were significantly (FDR < 0.1) enriched in GFW (Fig. 8a). Furthermore, gene co-expression modules were also significantly enriched for GWAS signals of all six traits (Fig. 8c). For instance, the gene co-expression module coloured yellow, which was significantly enriched in immune responses and the regulation of canonical Wnt signalling pathway (Fig. 7c), was significantly (FDR < 0.1) enriched for GWAS signals of MFD, CVFD, and CN (Fig. 8c).

By comprehensively integrating GWAS [40], the transcriptome data from this study, the sheep expression atlas [41, 42], and the human GWAS atlas [43, 44], we proposed the most promising candidate genes for each of the six traits (Additional file 17: Table S10). For instance, the top SNP of *SPHK1* explained 0.44% of the genetic variance (the fourth QTL region regarding the explained genetic variance) in CVFD and its expression level gradually increased during hair follicle development. *SPHK1* was specifically and highly expressed in the immune system and was strongly associated with immune-related traits such as the reticulocyte fraction of red blood cells in humans (Fig. 8d). The top SNP of *GHR* explained 0.76% (the third QTL region) and 0.39% (the ninth QTL region) of the genetic variance in CN and MSL, respectively. *GHR* was gradually downregulated during hair follicle development. It was specifically expressed in liver and was significantly associated (FDR < 0.05) with metabolism-related traits such as trunk fat-free mass in humans (Fig. 8e). *PPP1R27* showed the highest expression level at E105 compared to other stages, and its top SNP explained 0.36%, 0.37%, 0.24%, and 0.22% of genetic variance in LW, MFD, GFW, and CV, respectively. It was specifically expressed in muscle and was significantly associated with hair colour and haemoglobin levels in humans (Fig. 8f). The explained genetic variance, expression patterns across tissues, and phenome-wide association study (PheWAS) results of *CSRP2* for MFD, *EEF1A2* for MSL, and *PTPN1* for GFW are shown in Additional file 18: Fig. S8. In summary, the candidate genes discovered here showed specificity for developmental stage and tissue type, and their orthologues were associated with similar complex traits in humans.

## Discussion

The elucidation of the morphology and molecular mechanisms underlying the normal development of sheep hair follicles expands our understanding of hair growth biology and the genetic basis of wool traits. In this study, we first explored the morphogenesis of hair follicles using the H&E staining approach across six developmental stages, and demonstrated the asynchronous development of sheep hair follicles. Sheep and mouse exhibit the similar anatomical structure of the primary hair follicles, including the de novo formation of wool placode, dermal condensation, and the thickening of epidermis and dermis [4, 45]. However, sheep has secondary hair follicles, sweat glands, and postnatal hair growth cycles, while mouse lacks them. This indicates that although mammals share similarity during hair follicle development, sheep might exhibit distinct morphology and regulation mechanisms [46].

We demonstrated that stage-specific PCGs, non-coding RNAs, and DNA methylation play a critical role in hair follicle development. We discovered several stage-specific TFs, such as KLF4, LEF1, HOXC13, RBPJ, VDR, RARA, and STAT3, associated with hair follicle development and growth [24–30]. Functional enrichment analysis indicated that stage-specific genes were significantly enriched in signalling, cell migration, and aggregation, highlighting the central roles of intercellular crosstalk and dynamic cell rearrangement in the hair morphogenesis. Specifically, it has been demonstrated that the hair follicle fate was regulated by the canonical Wnt/β-catenin signalling [47], cellular differentiation by BMP signalling [48], and dermal papilla cell proliferation by Notch signalling [49].

We found that stage-specific genes (e.g. *IFN*, *CD40* and *TGFBR3*) and co-expression modules were significantly enriched in the immune system. Previous studies proposed that hair follicles had an immune capacity in the growth stage of hair cycle, characterized by the downregulation of major histocompatibility complex (MHC) class I and the upregulation of potent immunosuppressants in mammals [50]. This hair follicle immune capacity is also regulated by pathways like NF-kappa B, CD antigens, interleukins (IL), TNFs, and IFNs related [51]. The collapse of this hair follicle immune capacity has been proposed to initiate the loss of hair as seen in patients with the autoimmune disease alopecia areata (AA) [52, 53].

According to the biological hypothesis of ceRNA [54], circRNAs and lncRNAs regulate the expression of target genes by modulating miRNAs [55, 56]. In this study, by constructing ceRNA interaction networks using the stage-specific PCGs, miRNAs, circRNAs, and lncRNAs, we found that several circRNAs and lncRNAs regulated the expression of PCGs via modulating stage-specific miRNAs. We also found that a single miRNA can simultaneously target multiple circRNAs and lncRNAs, indicating that miRNA plays a central role in this interaction network by supplying multiple intermediate bridges linking circRNAs/lncRNAs to PCGs [57].

Identifying the causal genes and tissues for complex phenotypes contributes to animal breeding. Recent studies integrated various types of data from a wide range of tissues, such as gene expression [17, 58], DNA methylation [59], chromatin states [60, 61], histone modifications [62], and expression quantitative trait loci (eQTL) [63], with GWAS summary statistics to identify tissues and cell types that were relevant with complex traits and diseases. Here, by integrating developmental stage-specific PCGs, miRNAs, circRNAs, lncRNAs, and DNA methylation regions as well as gene co-expression modules with GWAS signals of wool and growth traits in sheep [40], we expanded our insights into the genetic architecture underlying such complex traits. However, functional experiments (e.g. gene editing) will be required to validate the candidate genes of wool traits identified in this study, and to assess their usefulness in animal breeding. In addition, the findings here can be further incorporated into genomic prediction models as biological priors, such as GFBLUP [64], for improving the prediction accuracy and for fine-mapping causal genes [17]. In future studies, additional omics data (e.g. ChIP-Seq, ATAC-Seq, Hi-C, and single-cell RNA-seq) from more tissue types (e.g. immune tissues) should be included to identify the molecular drivers underlying hair follicle development. Overall, the increasingly comprehensive functional annotations of genome will enable us to better elucidate the genetic basis of complex trait variation and to enhance the genetic improvement program in livestock [65].

## Conclusions

In summary, we here characterized the global changes of the whole transcriptome and DNA methylation across six developmental stages of hair follicle in sheep. We identified the key molecular features that were significantly associated with hair follicle morphogenesis, and highlighted the complexity of the regulatory networks of PCGs and non-coding RNAs during the hair follicle development. Through integrating these findings with GWAS of wool traits, we provided novel insights into the genetic and biological mechanisms underpinning such traits in sheep. These datasets and findings provided a valuable resource for understanding the biology of hair follicle development and for interpreting the genetic basis of skin-relevant traits in mammals.

## Methods

### Animal and tissue collection

All the experimental animals were Subo Merino sheep obtained from Xinjiang Fine Wool Sheep Breeding Farm (Xinjiang, China). Subo Merino is a sub-type of Merino, which was bred in 2014 in China by crossing Australian Merino (paternal) with the Chinese Merino (maternal) [66]. Eighteen healthy Subo Merino ewes (2–3 years old; MFD, 18.1 ± 0.5 μm) were artificially inseminated with fresh sperm from a Subo Merino ram (3 years old; MFD, 19.0 ± 0.4 μm) and then managed at the same flock. The pregnant ewes were housed indoor for a 7-day “settling-in period” prior to being euthanized (electrocution followed by exsanguination). The embryos were collected in pregnant ewes at four embryonic days (i.e. E65, E85, E105, and E135). The skin tissues were collected immediately after euthanasia. The skin tissues of postnatal lambs were collected in vivo with approximately 2 cm^2^ × 3 mm deep at P7 and P30. The wound was recovered in 2 weeks with care. Three biological replicates were generated for each of six developmental stages, and the body weight and length of all these 18 individuals were measured.

All eighteen skin tissues were collected from the right mid-side regions behind the shoulder blade of each individual and rinsed in 1 × phosphate-buffered saline (PBS). Each skin tissue was divided into two parts: One was cut into a strip and fixed in 4% paraformaldehyde at 4 °C for ~ 1 week before H&E staining. The H&E staining was performed according to the classic method [67]. The skin samples were then made into paraffin sections. We selected three horizontal sections from each individual and 10 fields of view for each section, to count the average number of PF and SF by taking pictures with the same magnification using the electron microscope. The remaining skin samples were minced and snap-frozen in liquid nitrogen for the subsequent RNA extraction.

### Library construction and sequencing

The total RNA from the 18 skin tissues was extracted using TRIzol reagent (Thermo Fisher Scientific, Waltham, MA, USA). All samples had high-quality RNA with RNA integrity number (RIN) > 8.0. For mRNA and lncRNA, the strand-specific sequencing libraries were constructed using the ribosomal RNA (rRNA) removal method following a previously described protocol [68]. For circRNA, the strand-specific sequencing libraries were constructed using rRNA-depleted and RNase R-digested methods according to the previous protocol [69]. For miRNAs, the RNA molecules in a size range of 18–30 nt were enriched from total RNA by the polyacrylamide gel electrophoresis (PAGE). The 3′ adaptors were then added, followed by the enrichment of RNAs with length of 36–44 nt and the ligation of 5′ adaptors to the RNAs. The ligation products were reverse-transcribed by PCR amplification. All of the above libraries were sequenced on the Illumina NovaSeq6000 platform, which generated 150-bp paired-end (PE150) reads.

For MeDIP-Seq, the DNA libraries were prepared with a TruSeq DNA ample preparation kit (Illumina, San Diego, CA, USA). In brief, genomic DNA was extracted with a DNeasy blood & tissue kit (Qiagen, Hilden, Germany). DNA (3 μg) was sonicated in the range of 100–500 bp. Subsequently, DNA underwent the end-repair, the generation of 3′-dA overhangs, and adaptor ligation steps using a Paired-End DNA Sample Prep kit (Illumina, San Diego, CA, USA). DNA was then recovered by AMPure XP Beads and used for MeDIP using the Magnetic Methylated DNA Immunoprecipitation Kit (Diagenode, Denville, NJ, USA) following the manufacturer’s protocol. Adaptor-mediated PCR was performed to amplify the enriched fragments, and the library was sequenced on an Illumina HiSeq2500 PE150 platform.

### Pre-processing sequence data

All raw reads were quality-tested with FastQC v. 0.11.8 (http://www.bioinformatics.babraham.ac.uk/projects/fastqc/). For the mRNAs and lncRNAs, clean reads were obtained by removing adaptor and low-quality reads with Seqtk [70]. The rRNA-free reads were mapped to the sheep reference genome (Ensembl Oar_v3.1) using Hisat2 v. 2.4 [71]. Stringtie v. 1.3.1 [72, 73] was used to count the fragment within each gene. The expression of PCGs and lncRNAs was normalized as fragments per kilobase of exon model per million mapped fragments (FPKM). To compare the newly built Oar_rambouillet_v1.0 with Oar_v3.1, we also mapped all 18 RNA-seq datasets to Oar_rambouillet_v1.0 using the same pipeline and found similar results in terms of mapping rates, gene expression, and stage-specific expression (Additional file 19: Table S11 and Additional file 20: Fig. S9). LncRNAs were defined as novel transcripts using the following filters: length ≥ 200 bp; number of exons ≥ 2; ORF ≤ 300 bp; have no or weak protein-coding ability (CPC score < 0 [74] & CNCI score < 0 [75] and no significant similarity with Pfam database [76]). Gffcompare v.0.9.8 [77] was used to compare lncRNAs derived from the current RNA-seq datasets with the known lncRNAs in NONCODE v5. The genes transcribed within 10 k bp up- and downstream of an lncRNA were considered as its *cis*-acting target genes. The *trans*-acting target genes of lncRNAs were predicted using RNAplex software [9].

For the circRNAs, clean reads were obtained by fastp v. 0.18.0 [78]. Reads were then aligned to the reference genome by Hisat2 v. 2.4 [71], and those with full length mapped were discarded. Next, from the unmapped reads, we extracted 20-nt from both ends and aligned them independently to find unique anchor positions within spliced exons by Hisat2 v. 2.4 [71]. Anchors aligning in the reverse orientation (head-to-tail) indicated circRNA splicing. They were then subjected to CIRI [79] to identify the circRNAs. The expression of circRNAs was quantified using the number of spanning back-spliced junction reads and normalized as spliced reads per billion mapping (SRPBM) [80].

For the miRNAs, clean tags were obtained by FASTX-Toolkit v. 0.0.13 [81] and aligned with small RNAs in the GeneBank (Release 209.0) and Rfam (Release 11.0) [82] databases to identify and remove rRNA, scRNA, snoRNA, snRNA, and tRNA. All clean tags were aligned to the sheep reference genome (Ensembl Oar_v3.1). The tags mapped to exons or introns from mRNA degradation and repeat sequences were removed. All clean tags were then searched against the miRBase (Release 22.1) [20] database to identify known miRNAs. The expression of miRNAs was calculated and normalized to counts per million (CPM). The target genes of miRNAs were predicted using three approaches, including mireap v. 0.20 [83], miRanda v. 3.3a [84], and TargetScan v. 7.0 [85, 86], and those detected by all three approaches were chosen for downstream analysis.

For the MeDIP-Seq data, raw reads were first processed to filter out low-quality reads that containing more than 5 “N”s or over 50% of the sequence with low-quality value (Phred score < 5) using FASTX-Toolkit v. 0.0.13 [81]. The clean reads were aligned to the sheep reference genome, allowing up to two mismatches, using Bowtie v. 0.12.8 [87]. Peak calling were conducted using MACS v. 2.1.1 [88]. Peaks detected in individual samples from the same developmental stage were merged using BEDTools [89]. Genomic features were annotated in the R package ChIPseeker [90]. Promoters were defined as 1500 bp up and 500 bp downstream from the TSS of each gene. To evaluate the enrichment of methylation peaks, the fold enrichment ratio was calculated as the MeDIP-Seq counts relative to expected background counts *λ*_local_ [88].

### Identification and annotation of stage-specific molecular features

Stage-specific PCGs (FDR < 0.05), lncRNAs (FDR < 0.05), circRNAs (*P* < 0.01), and miRNAs (*P* < 0.01) were identified between one stage and others using the R package edgeR [91]. Stage-specific MRs (*P* < 0.01) were identified using the R package DiffBind [92]. Stage-specific PCGs and circRNAs were separately clustered with the R *k*-means function where *k* = 6 within the cluster package according to the Euclidean distance. All functional enrichment analyses were conducted for each stage-specific gene types using the R package clusterProfile [93]. The edgeR package in R [48] was used to calculate the fold changes of PCGs in each stage compared to the other stages. These fold changes were used as input data in the GSEA. GSEA was performed to establish whether a set of genes in specific GO terms were significantly differed from the other stages using the R package GSVA [94, 95], together with the annotated gene sets C5 v. 7.1 downloaded from the MsigDB database [96]. Significantly enriched gene sets (FDR < 0.05) were then ranked by the consensus score [97]. The top five representative gene sets (FDR < 0.05) with the largest consensus scores were selected for each stage and visualized with the R package pheatmap [98]. The sequence motif enrichment analysis of promoters of stage-specific PCGs was conducted by MEME v. 5.3.3 [99], based on the JASPAR (2020) core non-redundant vertebrate motifs from Tomtom [99, 100].

### Regulatory network construction

A hypergeometric test was used to determine whether stage-specific PCGs were significantly (FDR < 0.05) enriched in miRNA or lncRNA targets. The regulatory networks of target genes of top three significantly (FDR < 0.05) enriched lncRNAs and miRNAs were visualized using Gephi v. 0.9.2 [101].

The ceRNA network was constructed according to the following steps: (1) All stage-specific transcripts were selected and the expression correlation between PCG-miRNA or lncRNA-miRNA or circRNA-miRNA was evaluated using the PCC. Pairs with PCC < − 0.7 were selected as negatively co-expressed pairs. (2) Among all lncRNA-PCG and circRNA-PCG pairs, those with PCC > 0.7 were selected as positively co-expressed pairs. (3) The hypergeometric test was used to determine whether the common miRNA sponges between the two genes were significant. Pairs with *P* < 0.05 were selected. (4) The ceRNA network was constructed by assembling all co-expressed competing triplets, which were identified in (3), and visualized using Gephi v. 0.9.2 [101].

### Weighted gene co-expression network analysis (WGCNA)

An unsigned gene co-expression network was constructed using the R package WGCNA v. 1.12.0 [62]. Briefly, 29,616 PCGs and non-coding RNAs were used for the analyses. They all had expression > 0.1 (FPKM for PCG and lncRNA, SRPBM for circRNA, CPM for miRNA) in ≥ 12 samples. The normalized matrix was transformed to a matrix of Pearson correlations between gene pairs which, in turn, was converted to an adjacency matrix. To identify highly co-expressed gene modules, genes with similar expression patterns (*r* > 0.9) were clustered with a dynamic hybrid cutting algorithm. Eigengenes for these modules were defined as the first principal component of the corresponding expression matrix and were associated with all six hair follicle developmental stages.

### GWAS signal enrichment analysis

The details of the weighted single-step genome-wide association study (WssGWAS) for five wool traits and one growth trait including MFD, CVFD, CN, MSL, GFW, and LW in Merino sheep was described previously [40]. Briefly, the GWAS population consisted of 7135 individuals (aged at 15 months) with phenotype data, among which 1217 had imputed high-density (HD) genotype data (*n* = 372,534) [40]. A sum-based marker-set test method was applied using the QGG package in R [17, 102] for GWAS signal enrichment analyses across stage-specific molecular features and gene co-expression modules:

1 $$ {\boldsymbol{T}}_{\boldsymbol{sum}}=\sum \limits_{\boldsymbol{i}=\mathbf{1}}^{{\boldsymbol{m}}_{\boldsymbol{g}}}{\boldsymbol{b}}^{\mathbf{2}} $$

where *T*_*sum*_ represents the summary statistics for each molecular feature, ***m***_***g***_ is the number of SNPs overlapping the molecular feature, and ***b*** is the SNP effect from GWAS. The marker-set sizes and LD patterns among markers were controlled by applying a genotype cyclical permutation strategy as described previously [103, 104]. To obtain empirical *P* values for the association of a molecular feature with a complex trait, the permutation procedure was repeated 10,000 times and a one-tailed test was applied based on the proportion of random summary statistics greater than that observed [17, 102]. The *P* values were corrected for multiple testing using the FDR method. FDR < 0.1 was considered significant.

### Detection of candidate genes of wool and growth traits with multiple data sources

To detect candidate genes of wool and growth traits in sheep, we first focused on the stage-specific genes that were located in the top 50 ranked QTLs in terms of their explained genetic variance for each trait. The proportion of genetic variance explained by SNPs of candidate genes is derived from WssGWAS [40, 105]. To detect whether candidate genes show tissue-specific expression in a wide range of tissues and cell types, the gene expression estimates (transcripts per million, TPM) of 500 ovine samples were downloaded from the sheep gene atlas as reported by Clark et al. [41, 42]. They comprised 87 tissue and cell types with varying numbers of animals per tissue type. According to the known tissue biology [41], the samples were classified into 13 organ systems. The details are described in Additional file 21: Table S12.

The PheWAS has been widely used to associate a genetic variant with many phenotypes to explore its pleiotropic effect on complex traits [43]. To detect whether the human orthologues of candidate genes detected for wool and growth traits in sheep here are associated with similar traits in humans, a PheWAS analysis was conducted for each candidate gene based on the GWASATLAS database [43, 44]. We used the GWAS summary statistics of 586 complex phenotypes from 159 publicly available human GWAS (the total sample size of each study > 2000). We considered genes with adjusted *P* values (FDR) less than 0.05 as significant. The complex traits were classified into 12 trait domains based on the known biology [106, 107]. The details are described in Additional file 22: Table S13.

## Supplementary Information

**Additional file 1: Fig. S1**. The global study design. Grey boxes represent 18 samples collected from skin tissue at six developmental stages (three biological replicates per stage) of hair follicle in sheep. The H&E staining method is used to observe the morphology of each sample. Orange boxes are for data generation, including strand-specific RNA-Seq for mRNA, lncRNA, circRNA and miRNA, as well as MeDIP-seq for DNA methylation in each of 18 skin samples. Green boxes show the major bioinformatics and statistical analyses involved in this study for functional annotation of stage-specific molecular signatures. Blue boxes describe other resources used for detecting candidate genes of wool traits in sheep. Pink boxes outline the main objective of this study, which is to reveal the genetic and biological basis of hair follicle development in sheep.

**Additional file 2: Table S1**. Mapping summary of all sequence data in this study.

**Additional file 3: Table S2**. Comparison of expressed genes detected in this study with known genes in sheep reference genome.)

**Additional file 4: Table S3**. The distribution of DNA methylation peaks among different genomic features.

**Additional file 5: Fig. S2**. Principal component analysis (PCA) of samples based on the expression levels of all four gene types including protein-coding genes (PCGs), lncRNAs, circRNAs and miRNAs.

**Additional file 6: Fig. S3**. General characteristics of circRNAs in sheep skin. a, Genomic origin of circRNAs in sheep skin. b, Distribution of parental genes (hosting genes) encoding different numbers of circRNAs in sheep skin.

**Additional file 7: Fig. S4**. Identification of stage-specific molecular signatures during sheep hair follicle development. a, b, c, d, e, and f are numbers of stage-specific PCGs (FDR < 0.05), lncRNAs (FDR < 0.05), circRNAs (*P* < 0.01), miRNAs (*P* < 0.01), methylation regions (MRs) (*P* < 0.01) and genes overlapped in MRs, respectively. Solid lines: upregulation; dashed lines: downregulation.

**Additional file 8: Table. S4**. Top five representative Gene Ontology (GO) terms for upregulated and downregulated genes at each developmental stage revealed by a gene set enrichment analysis (GSEA).

**Additional file 9: Table S5**. Motif enrichment analysis for promoters of upregulated and downregulated stage-specific genes.

**Additional file 10: Fig. S5**. Functional enrichment analysis of down-regulated circRNAs during sheep skin development. Heatmap shows the normalized expression of top 10 downregulated stage-specific circRNAs at each developmental stage. Bubble plot shows the top five enriched Gene Ontology (GO) terms (biological process; BP) for parental genes of downregulated stage-specific circRNAs.

**Additional file 11: Fig. S6**. Functional enrichment analysis of up- and down-regulated lncRNAs and miRNAs during sheep hair follicle development. Heatmaps show the normalized expression of top 10 upregulated and downregulated lncRNAs (a, b) and miRNAs (c, d) at each developmental stage. Bubble plots show the top five enriched gene ontology (GO) terms (biological process; BP) for targets of upregulated and downregulated lncRNAs (a, b) and miRNAs (c, d).

**Additional file 12: Table S6**. Number of significantly (FDR < 0.05) enriched lncRNAs and miRNAs.

**Additional file 13: Table S7**. Number of the negatively correlated (Pearson correlation coefficient (PCC) < -0.7) miRNA-targets pairs.

**Additional file 14: Table S8**. Number of the positively correlated (Pearson correlation coefficient (PCC) > 0.7) endogenous RNA (ceRNA) pairs targeted by a common miRNA (*P* < 0.05).

**Additional file 15: Fig. S7**. Gene co-expression modules determined by a weighted gene co-expression network analysis (WGCNA). a, Heatmap on the left shows the normalized gene expression of 15 modules. Top enriched Gene Ontology (GO) terms for each module are summarized on the right. b, Bar chart shows the numbers of annotated and unannotated genes in each module. c, Density chart shows the percentage of annotated and unannotated genes in each module. b and c share the same figure legend.

**Additional file 16: Table S9**. Significant GWAS signal enrichment results in wool and growth traits.

**Additional file 17: Table S10**. Summary of candidate genes for wool traits and live weight in Merino sheep.

**Additional file 18: Fig. S8**. Integrative analysis with multi-databases to detect candidate genes for wool traits and live weight in Merino sheep. a, *CSRP2*; b, *EEF1A2*; c, *PTPN1*. Plots (from left to right) show the genetic variance explained by SNPs of candidate gene (each dot is one SNP), the expression patterns of corresponding genes during sheep skin development, the expression patterns of corresponding genes across multi-tissues, and the phenome-wide association study (PheWAS) results for each candidate gene in humans, respectively.

**Additional file 19: Table S11**. Comparisons of the mapping statistics of 18 RNA-seq datasets between Oar_v3.1 and Oar_rambouillet_v1.0.

**Additional file 20: Fig. S9**. Comparison of gene expression for all 18 RNA-seq samples between Oar_v3.1 and Oar_rambouillet_v1.0. a. The Pearson correlation coefficient (PCC) of mean log_2_(count+ 1) is 0.95, *P*-value< 2.2e-16; b. The PCC of mean log_2_(FPKM+ 1) is 0.94, *P*-value < 2.2e-16. c and d The PCC of log_2_(count+ 1) and log_2_(FPKM+ 1) for each sample, respectively. e and f. Comparison of the stage-specific expression of PCGs between the two genome assemblies (Oar_v3.1 and Oar_rambouillet_v1.0).

**Additional file 21: Table S12**. Gene expression levels (transcripts per million, TPM) of six candidate genes in sheep gene expression atlas.

**Additional file 22: Table S13**. Phenome-wide association study (PheWAS) results for six candidate genes in humans.

## Data Availability

All raw data generated in this study were submitted to the National Center for Biotechnology Information Sequence Read Archive (NCBI SRA) database under BioProject Nos. PRJNA705405 [108], PRJNA705547 [109], PRJNA705552 [110], and PRJNA705554 [111]. The GWAS summary data used in the current study were obtained from [40]. The sheep expression atlas was obtained from [41, 42]. Human PheWAS data is available from [43, 44].

## Abbreviations

CeRNA: Competing endogenous RNA
circRNA: Circular RNA
CN: Crimp number
CNS: Central nervous system
CPM: Counts per million
CVFD: Coefficient of variation of fibre diameter
FPKM: Fragments per kilobase of exon model per million mapped fragments
GFW: Greasy fleece weight
GI Tract: Gastrointestinal tract
GO: Gene ontology
lncRNA: Long non-coding RNA
LW: Live weight
MeDlP-Seq: Methylated DNA immunoprecipitation sequencing
MFD: Mean fibre diameter
miRNA: MicroRNA
MRs: Methylated regions
MSL: Mean staple length
ncRNA: Non-coding RNA
PCA: Principal component analysis
PCC: Pearson correlation coefficient
PCG: Protein-coding gene
PheWAS: Phenome-wide association study
SRPBM: Spliced reads per billion mapping
TF: Transcriptional factor
TSS: Ttranscriptional start site
WGCNA: Weighted gene co-expression network analysis
WssGWAS: Weighted single-step genomic association study
