# Supplementary figures and images for: Comprehensive transcriptome and methylome analysis delineates the biological basis of hair follicle development and wool-related traits in Merino sheep
Source: BMC Biol. 2021 Sep 9;19:197. doi: 10.1186/s12915-021-01127-9 (PMC8427949; doi:10.1186/s12915-021-01127-9)

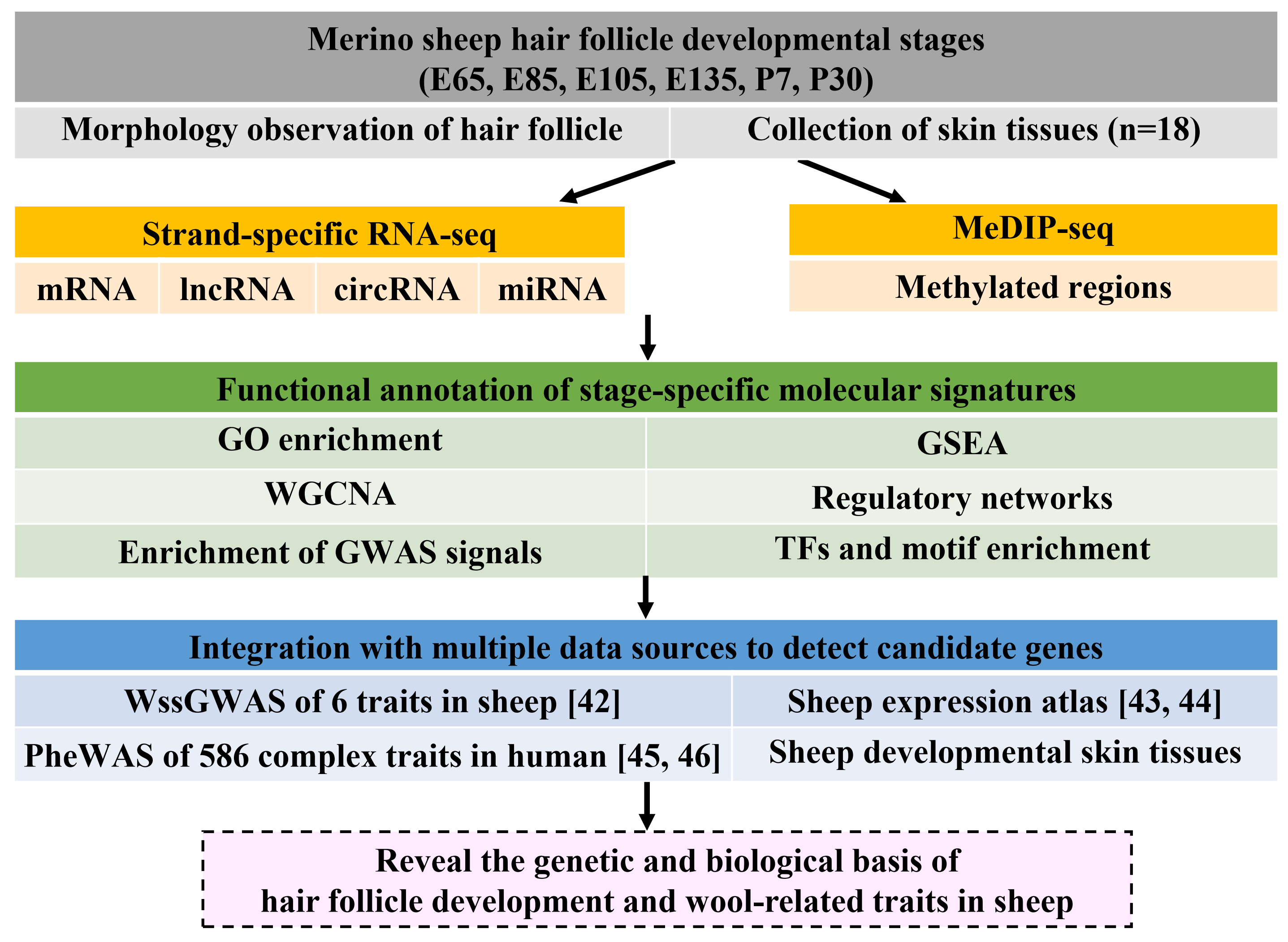

Supplement: Supplementary file 1 — Additional file 1: Fig. S1. The global study design. Grey boxes represent 18 samples collected from skin tissue at six developmental stages (three biological replicates per stage) of hair follicle in sheep. The H&E staining method is used to observe the morphology of each sample. Orange boxes are for data generation, including strand-specific RNA-Seq for mRNA, lncRNA, circRNA and miRNA, as well as MeDIP-seq for DNA methylation in each of 18 skin samples. Green boxes show the major bioinformatics and statistical analyses involved in this study for functional annotation of stage-specific molecular signatures. Blue boxes describe other resources used for detecting candidate genes of wool traits in sheep. Pink boxes outline the main objective of this study, which is to reveal the genetic and biological basis of hair follicle development in sheep. [file 12915_2021_1127_MOESM1_ESM.tiff]

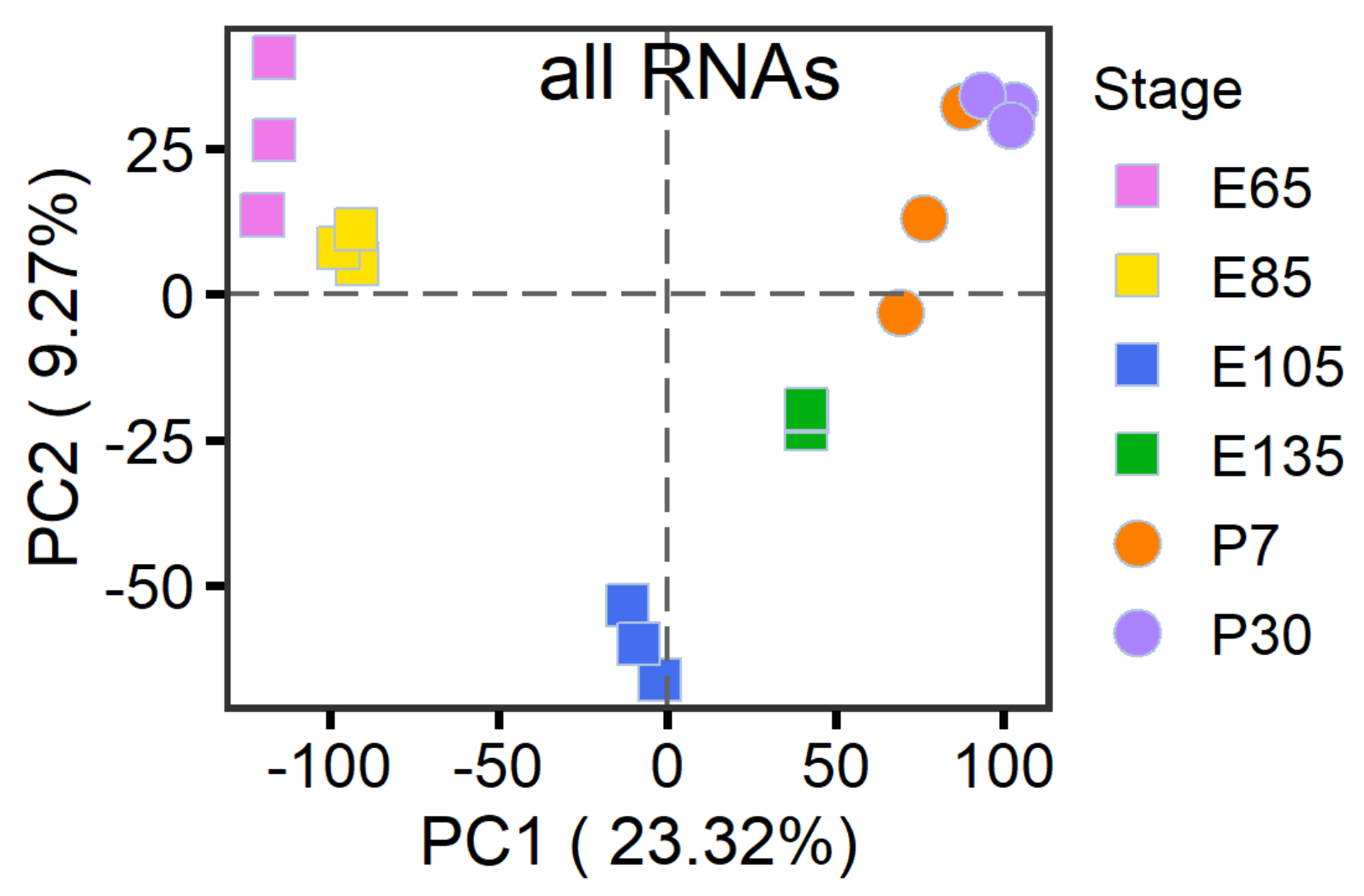

Supplement: Supplementary file 5 — Additional file 5: Fig. S2. Principal component analysis (PCA) of samples based on the expression levels of all four gene types including protein-coding genes (PCGs), lncRNAs, circRNAs and miRNAs. [file 12915_2021_1127_MOESM5_ESM.tiff]

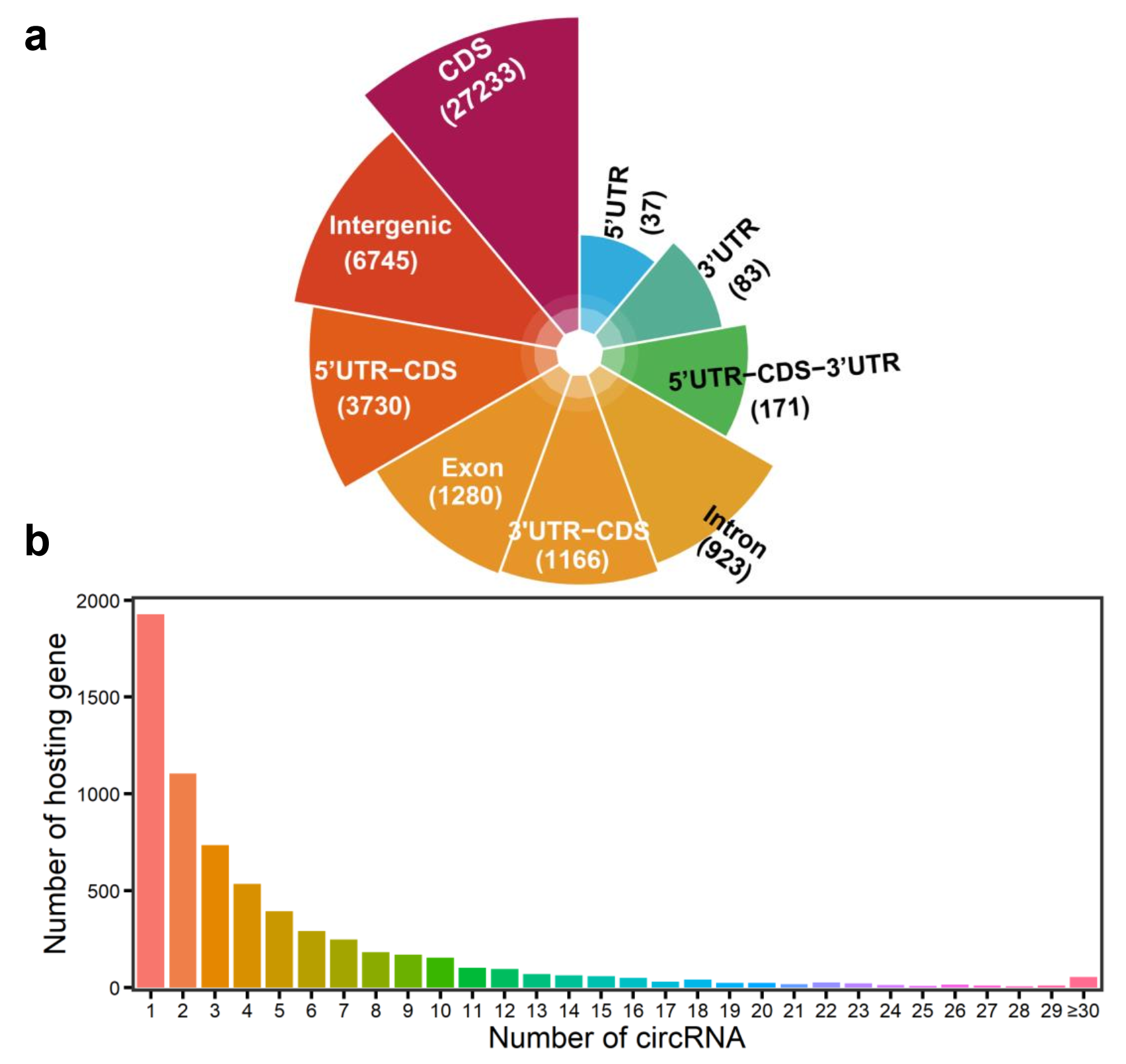

Supplement: Supplementary file 6 — Additional file 6: Fig. S3. General characteristics of circRNAs in sheep skin. a, Genomic origin of circRNAs in sheep skin. b, Distribution of parental genes (hosting genes) encoding different numbers of circRNAs in sheep skin. [file 12915_2021_1127_MOESM6_ESM.tif]

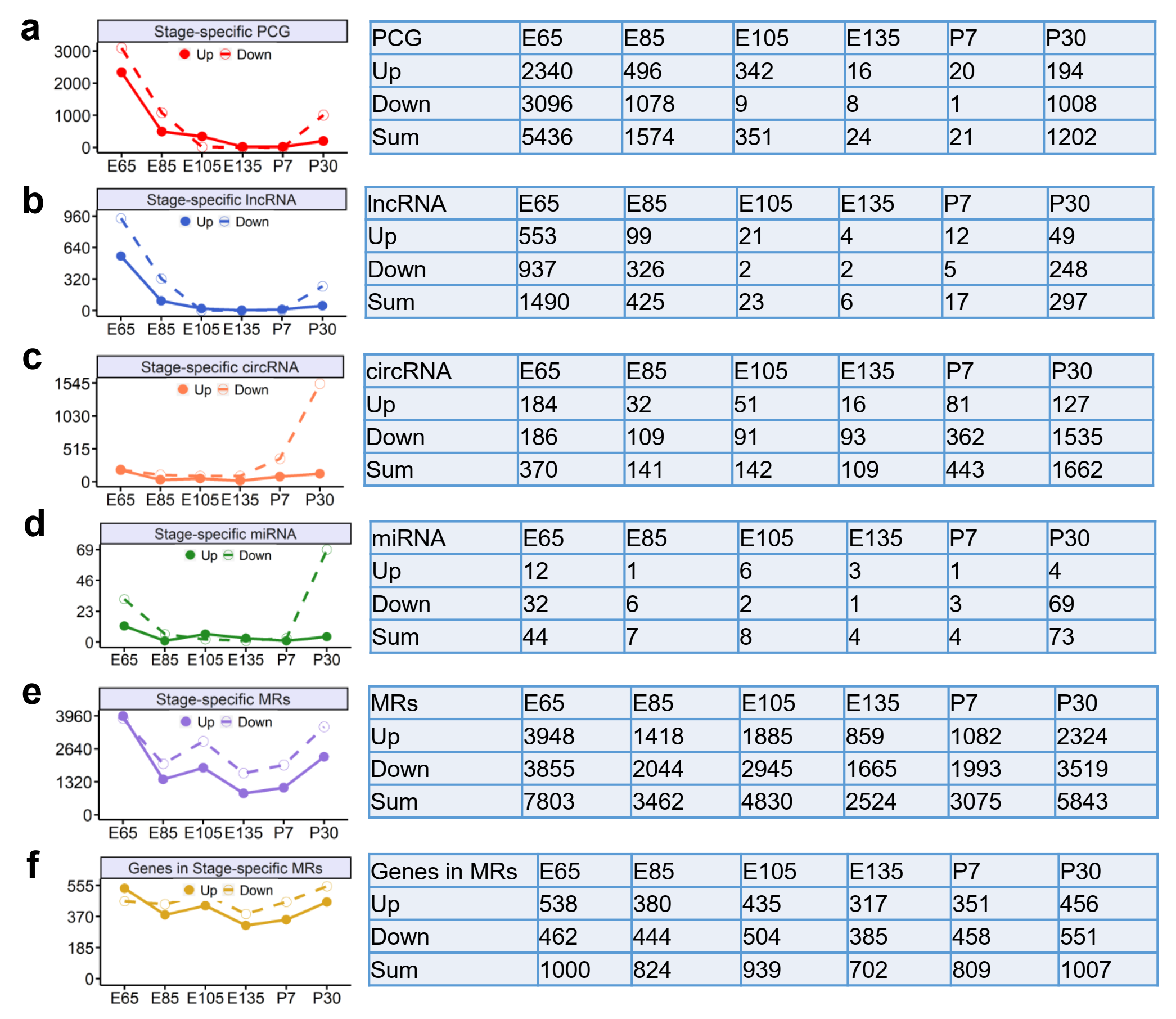

Supplement: Supplementary file 7 — Additional file 7: Fig. S4. Identification of stage-specific molecular signatures during sheep hair follicle development. a, b, c, d, e, and f are numbers of stage-specific PCGs (FDR < 0.05), lncRNAs (FDR < 0.05), circRNAs (P < 0.01), miRNAs (P < 0.01), methylation regions (MRs) (P < 0.01) and genes overlapped in MRs, respectively. Solid lines: upregulation; dashed lines: downregulation. [file 12915_2021_1127_MOESM7_ESM.tiff]

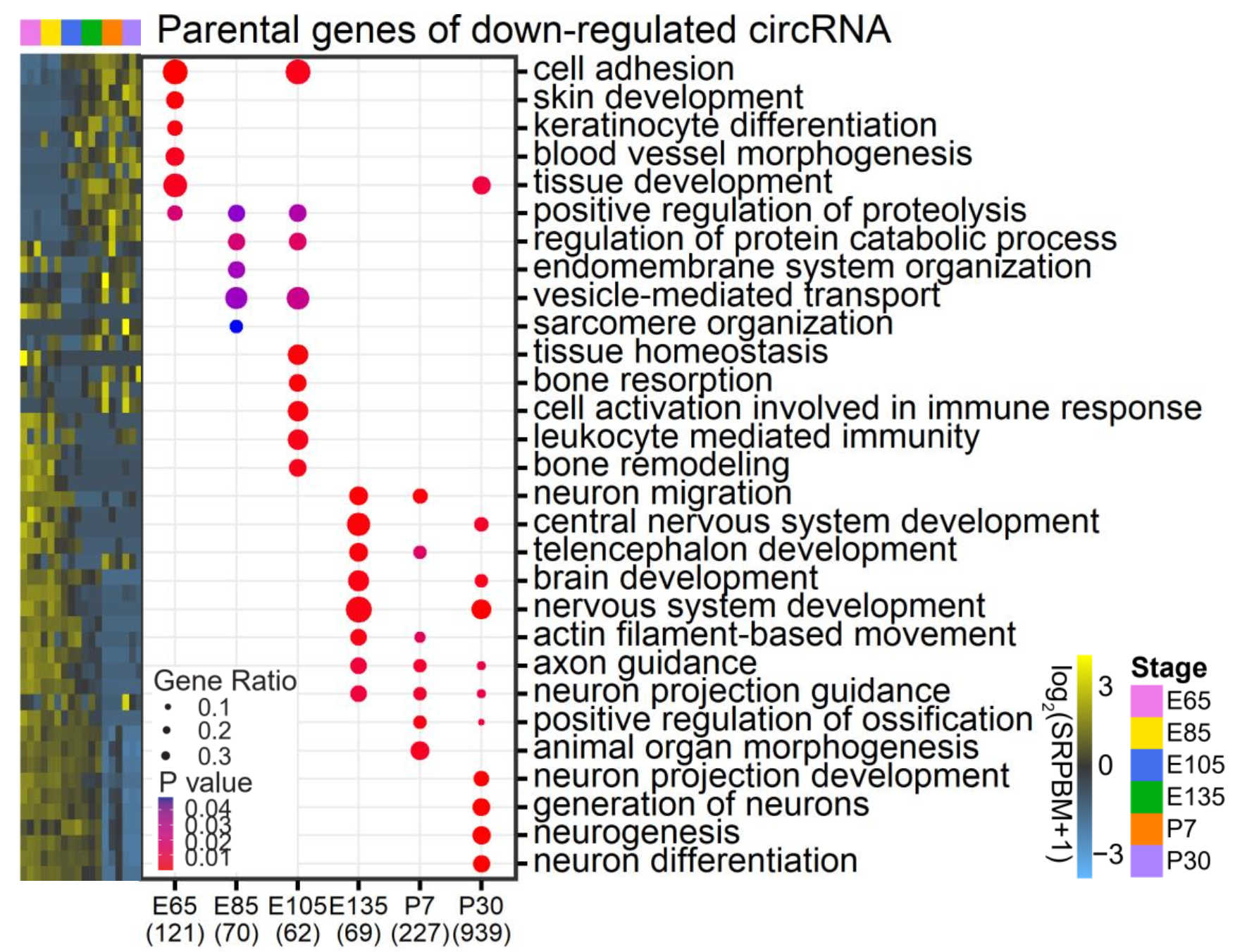

Supplement: Supplementary file 10 — Additional file 10: Fig. S5. Functional enrichment analysis of down-regulated circRNAs during sheep skin development. Heatmap shows the normalized expression of top 10 downregulated stage-specific circRNAs at each developmental stage. Bubble plot shows the top five enriched Gene Ontology (GO) terms (biological process; BP) for parental genes of downregulated stage-specific circRNAs. [file 12915_2021_1127_MOESM10_ESM.tiff]

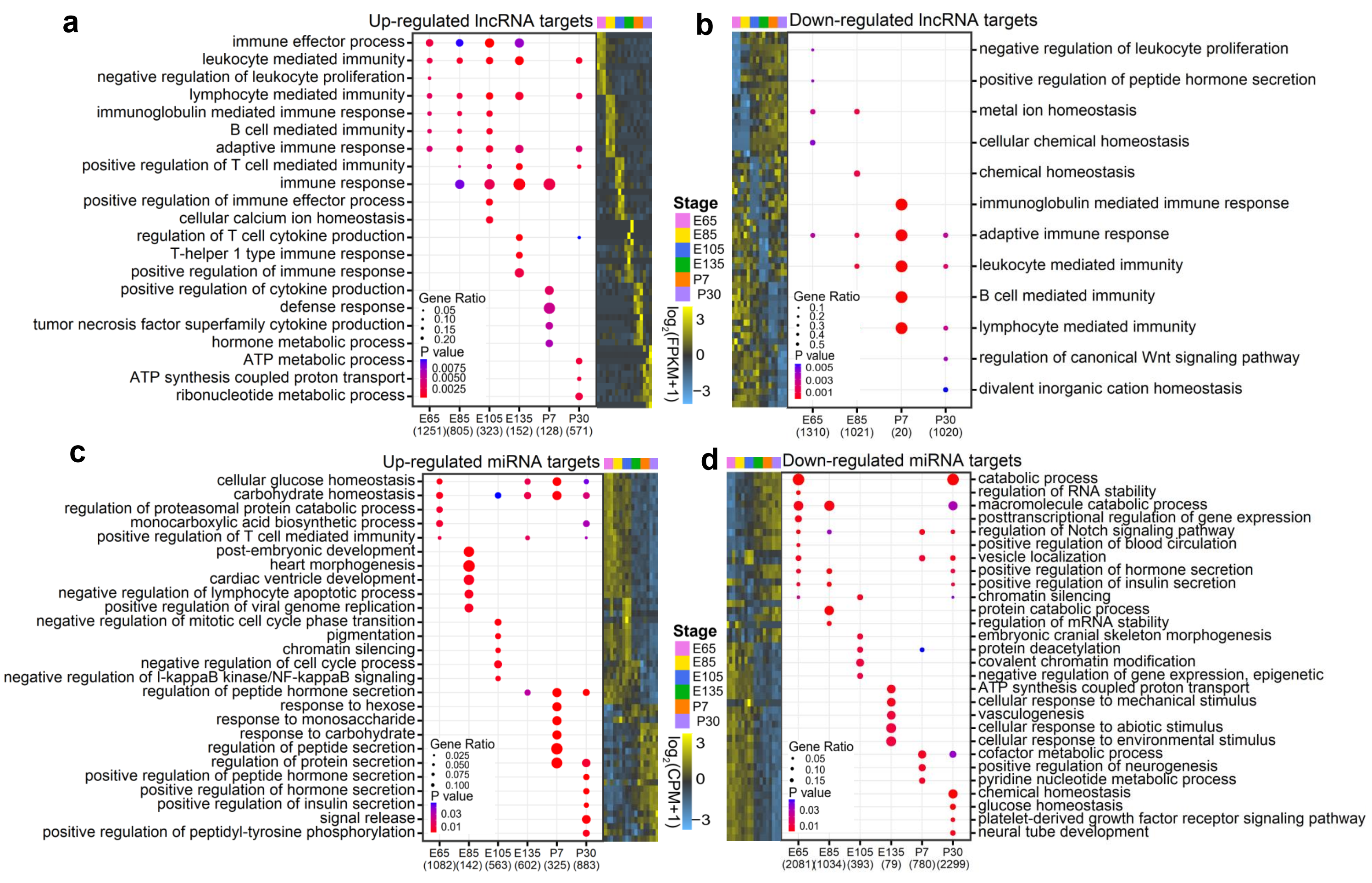

Supplement: Supplementary file 11 — Additional file 11: Fig. S6. Functional enrichment analysis of up- and down-regulated lncRNAs and miRNAs during sheep hair follicle development. Heatmaps show the normalized expression of top 10 upregulated and downregulated lncRNAs (a, b) and miRNAs (c, d) at each developmental stage. Bubble plots show the top five enriched gene ontology (GO) terms (biological process; BP) for targets of upregulated and downregulated lncRNAs (a, b) and miRNAs (c, d). [file 12915_2021_1127_MOESM11_ESM.tiff]

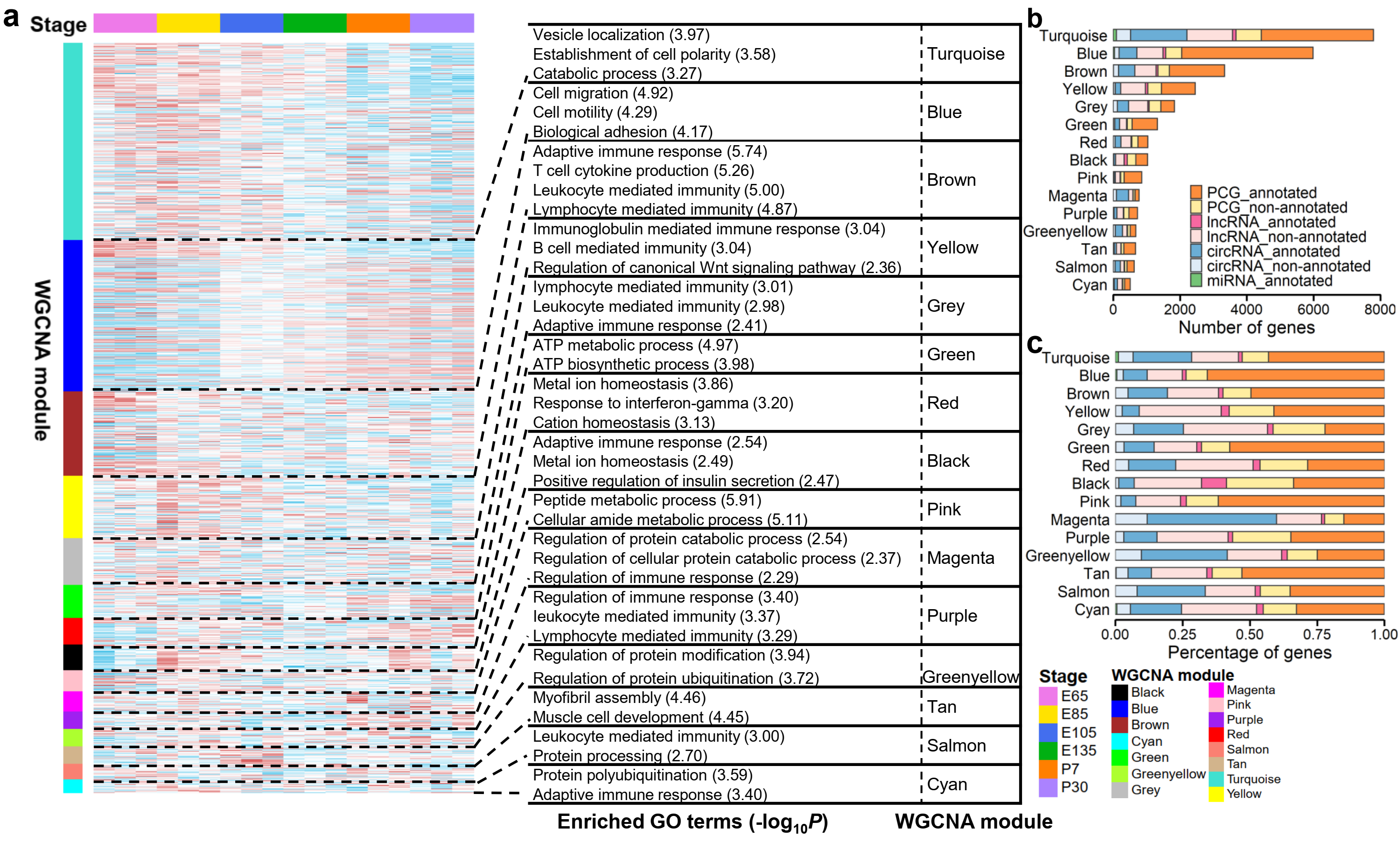

Supplement: Supplementary file 15 — Additional file 15: Fig. S7. Gene co-expression modules determined by a weighted gene co-expression network analysis (WGCNA). a, Heatmap on the left shows the normalized gene expression of 15 modules. Top enriched Gene Ontology (GO) terms for each module are summarized on the right. b, Bar chart shows the numbers of annotated and unannotated genes in each module. c, Density chart shows the percentage of annotated and unannotated genes in each module. b and c share the same figure legend. [file 12915_2021_1127_MOESM15_ESM.tiff]

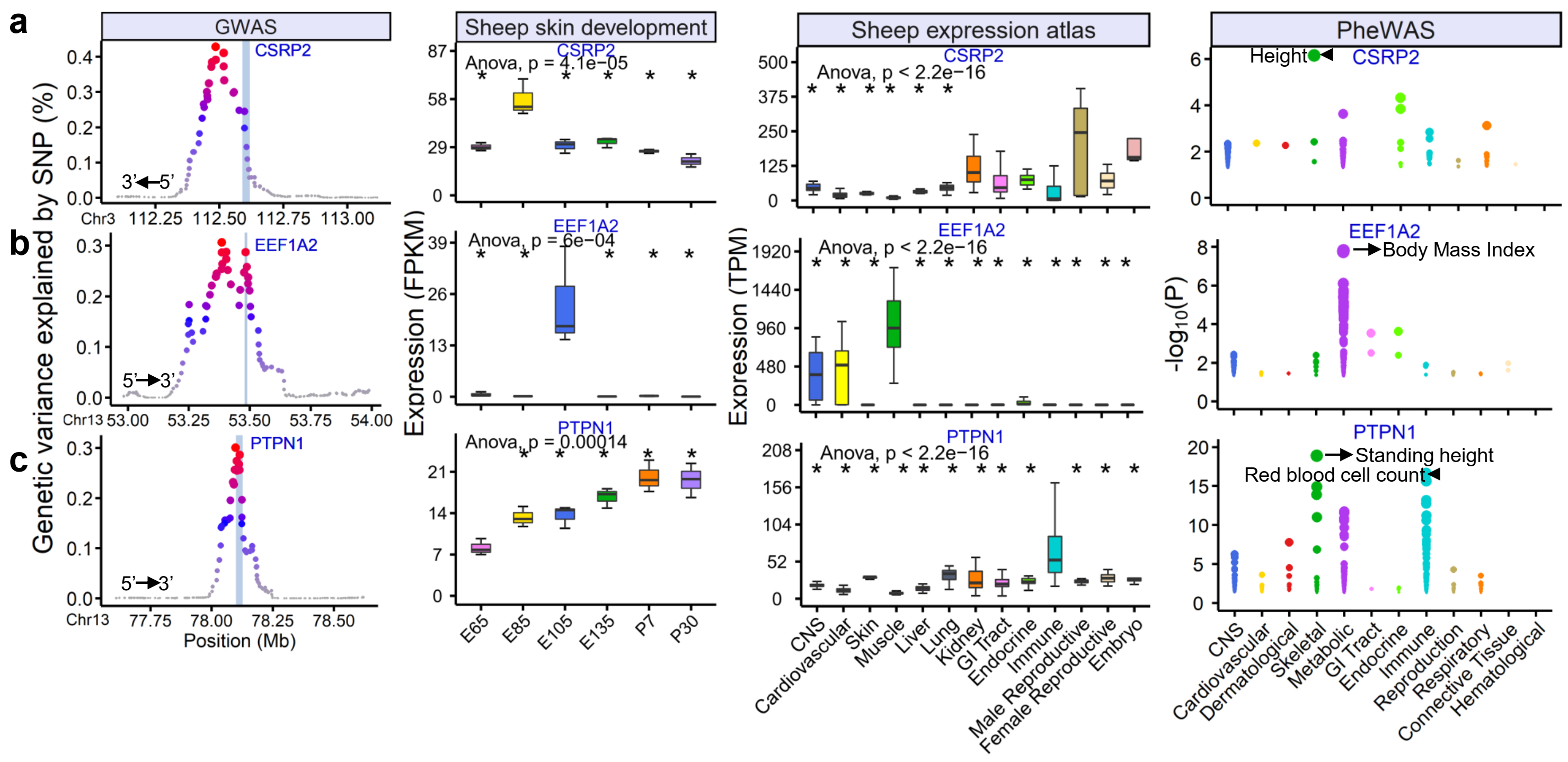

Supplement: Supplementary file 18 — Additional file 18: Fig. S8. Integrative analysis with multi-databases to detect candidate genes for wool traits and live weight in Merino sheep. a, CSRP2; b, EEF1A2; c, PTPN1. Plots (from left to right) show the genetic variance explained by SNPs of candidate gene (each dot is one SNP), the expression patterns of corresponding genes during sheep skin development, the expression patterns of corresponding genes across multi-tissues, and the phenome-wide association study (PheWAS) results for each candidate gene in humans, respectively. [file 12915_2021_1127_MOESM18_ESM.tiff]

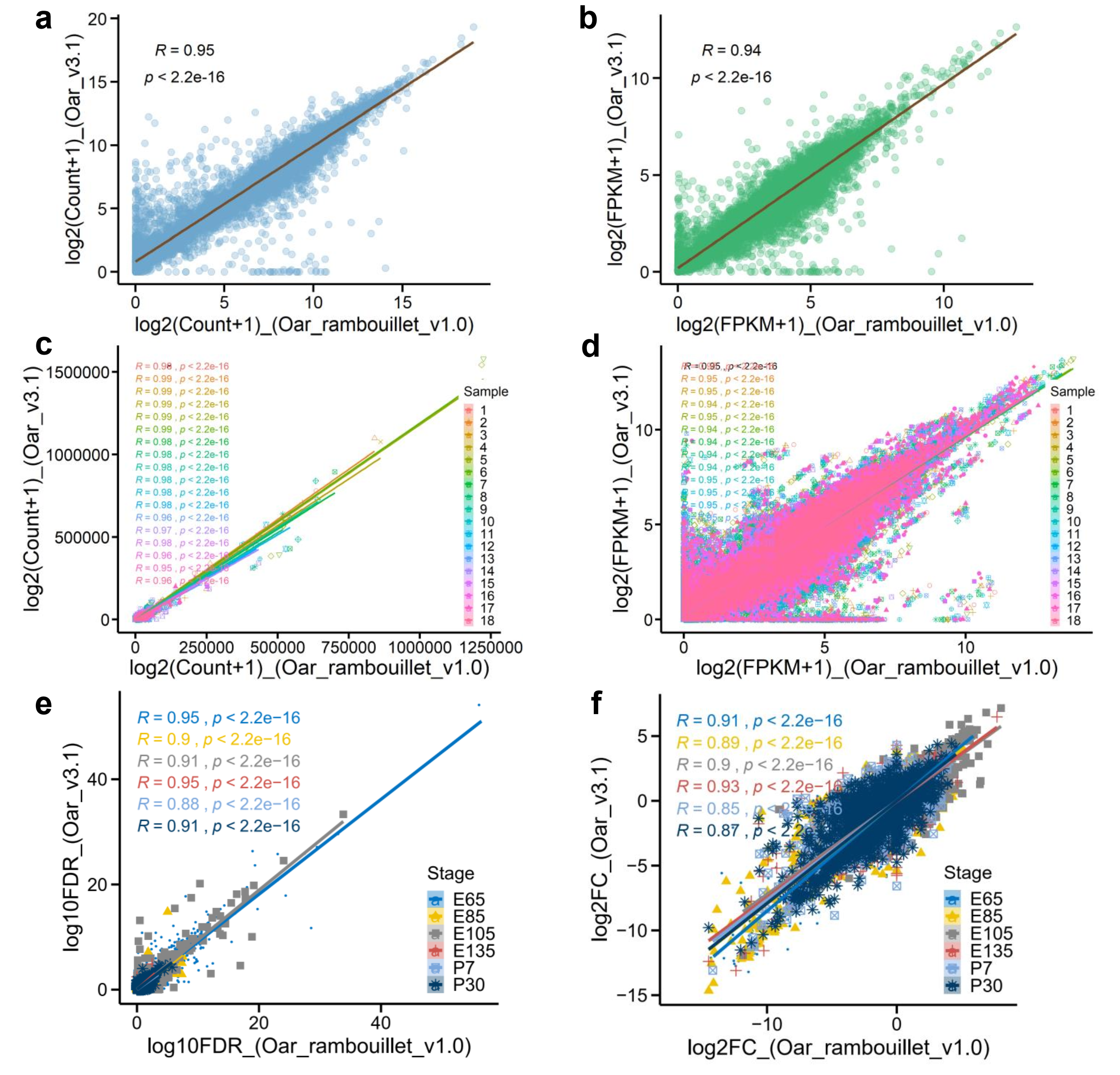

Supplement: Supplementary file 20 — Additional file 20: Fig. S9. Comparison of gene expression for all 18 RNA-seq samples between Oar_v3.1 and Oar_rambouillet_v1.0. a. The Pearson correlation coefficient (PCC) of mean log2(count+ 1) is 0.95, P-value< 2.2e-16; b. The PCC of mean log2(FPKM+ 1) is 0.94, P-value < 2.2e-16. c and d The PCC of log2(count+ 1) and log2(FPKM+ 1) for each sample, respectively. e and f. Comparison of the stage-specific expression of PCGs between the two genome assemblies (Oar_v3.1 and Oar_rambouillet_v1.0). [file 12915_2021_1127_MOESM20_ESM.tiff]
